# Surfactant-Free Microemulsion as Template for Stöber Silica Nanoparticle Synthesis

**DOI:** 10.1021/acsomega.6c08994

**Published:** 2026-10-13

**Authors:** Bastian Rödig, Lena Schmauser, Patrick Denk, Werner Kunz

**Affiliations:** † Institut für physikalische und theoretische Chemie, Universität Regensburg, D-93053 Regensburg, Germany; ‡ Lehrstuhl für Physikalische Chemie II und Institut für Physikalische Chemie, RWTH Aachen, D-52074 Aachen, Germany

## Abstract

Surfactant-free microemulsions (SFMEs) have emerged as promising structured reaction media for the synthesis of inorganic nanoparticles. Recent reports suggested that the ternary water (H_2_O)/ethanol (EtOH)/tetraethyl orthosilicate (TEOS) system forms SFME structures capable of directing silica particle formation through a self-templating mechanism. Here, we systematically investigate this hypothesis and evaluate the influence of SFME structuring on Stöber silica nanoparticle synthesis. Ternary and pseudoternary phase diagrams of H_2_O/EtOH/TEOS and H_2_O/EtOH/(oil + TEOS) systems were established and characterized by dynamic light scattering (DLS), small- and wide-angle X-ray scattering (SWAXS), and transmission electron microscopy (TEM). The H_2_O/EtOH/TEOS system was found to remain molecularly dispersed throughout the investigated monophasic region and showed no evidence of SFME aggregate formation. Consequently, silica particle formation in this system follows conventional Stöber-type kinetics rather than a self-templating mechanism. Nevertheless, synthesis at high water contents and low ammonia concentrations produced highly monodisperse silica nanoparticles in the low-nanometer size range and enabled controlled particle growth through regrowth steps. In contrast, genuine SFME systems containing toluene or 1-octanol exhibited pronounced mesoscale structuring in the pre-Ouzo and bicontinuous regions, which strongly affected nucleation and growth behavior. Time-resolved DLS measurements revealed induction periods and growth regimes characteristic of compartmentalized reaction environments, resulting in particle sizes ranging from a few nanometers to several hundred nanometers, depending on microstructure and oil composition. The results demonstrate that SFME structures do not template particle formation in the H_2_O/EtOH/TEOS system but can substantially modify Stöber kinetics when a genuine mesoscale organization is present. A mechanistic model is proposed in which diffusion of hydrolyzed silica species across the SFME interphase governs nucleation and growth, establishing interfacial mass transport as a key parameter for controlling silica nanoparticle formation in structured solvents.

## Graphical abstract

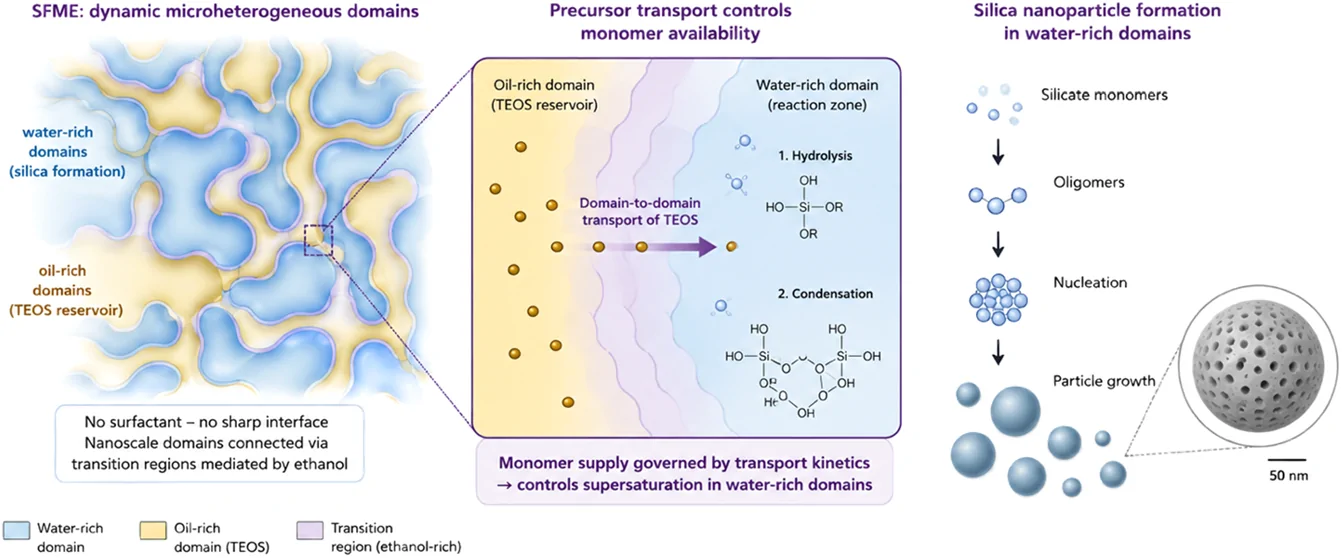

## 1 Introduction

Nowadays, the synthesis of well-defined colloidal particles with uniform composition and distinct surface properties attracts great attention. Especially nontoxic, sustainable, and cheap materials with interesting chemical, electronic, and optical properties are of high interest.^1^ One such material, silica, offers excellent possibilities to modify the surface and also shows little toxicity, which explains the already widespread usage of such materials.^2^

The most commonly known silica particle synthesis, often just called Stöber synthesis or sometimes SFB synthesis, goes back to 1968 when Stöber, Fink, and Bohn first reported the controlled growth of silica spheres in aqueous alcoholic solutions.^3^ This way, well-defined, highly monodisperse, uniform silica particles could be prepared. In the first 30 years since the paper was published, interest in such particles was limited. Only in the last 20 years have the synthesis and application of such particles become a growing focus of scientific publications.^4^

By variation of the initial reaction parameters, we can control the structure and size of the final particles. A few general statements can be made if the various reaction parameters are ranked according to the strength of their influence on the final particle size: temperature *T* > [H_2_O]/[TEOS] > [H_2_O]/[EtOH] > [NH_3_].^5^

Ternary mixtures consisting of two immiscible compounds and an amphiphile exhibit interesting structural phenomena.^6^ As indicated by their name, amphiphiles consist of both hydrophilic and hydrophobic moieties. Depending on the amphiphilicity of the molecule, the amphiphiles are either considered as a surfactant (surface active agent) or as a hydrotrope.^7^ Compared directly, the hydrophobic segment in hydrotropes is rather small, resulting in reduced amphiphilic character, meaning considerably higher amounts are necessary to achieve similar effects to surfactants.^8–11^ Analogous to water–surfactant-oil systems, ternary phase diagrams can be constructed for systems containing water, hydrotrope, and oil.

These ternary mixtures, showing different structured phases, partly similar to surfactant-containing mixtures, can be called surfactant-free microemulsion (SFME).^12,13^ Such structured solvents have attracted growing interest over the past decades due to their wide variety of possible applications.^14–18^ In SFMEs, the role of the amphiphilic component is covered by the hydrotrope, which offers several advantages over surfactant-based systems. They are usually more biodegradable, less toxic, exhibit less foaming capability, exhibit no liquid crystal formation, and are easier to remove.^15,19^ Not every ternary mixture containing water, oil, and hydrotrope is capable of forming structured SFMEs. Even though there is no comprehensive explanation yet, the choice of both oil and hydrotrope has a strong influence on the resulting structure.^20^

Further, the use of structured solvents appears to be a good way to perform template-based syntheses of both organic polymers^21,22^ and inorganic particles.^17,23–25^

Over the years, since the first publication on Stöber particles, many advancements have been made. Nevertheless, most of the processes used still rely on the use of ethanol as a main solvent, which is neither economically viable nor environmentally sustainable, as large quantities of organic solvent are wasted during the process. For better comparison, an overview of the ternary mixtures from important publications is presented in a ternary phase diagram (cf. Figure 1).

**Figure 1:**
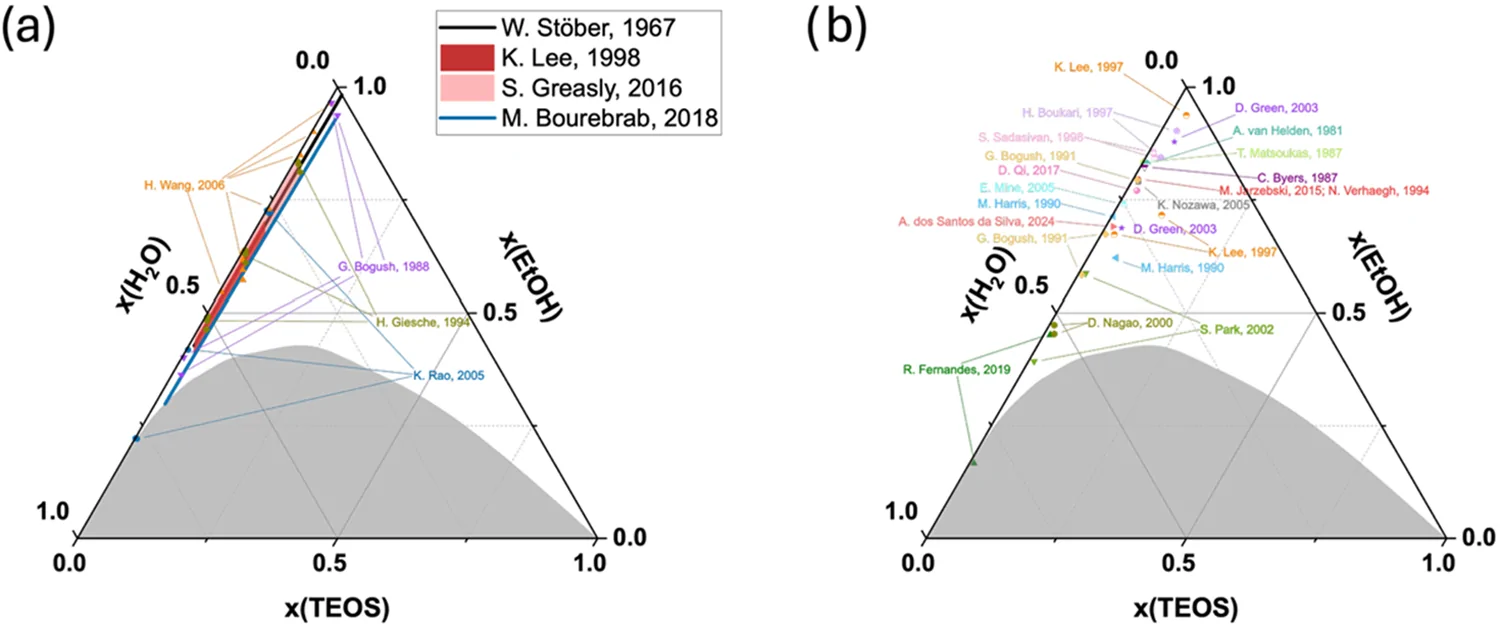
Phase diagrams of the ternary mixture of H_2_O/EtOH/TEOS expressed in molar fractions x. The positions in the ternary space of relevant publications on Stöber syntheses were plotted. (a) Highlighted areas correspond to screenings conducted by refs 3,26–28 . The single points were chosen as all references investigated rather different concentration ranges.^29–32^ (b) Shown references only conducted experiments on few points within the ternary phase diagram.^5,26,33–49^ Note that a majority of the conducted experiments are at reduced water concentrations. Enlarged versions can be viewed in the Supporting Information (SI).

Typically, the [TEOS]/[NH_3_] ratio does not exceed a value of 1 with few exceptions,^33,40,42,50^ and the majority of the syntheses used in the literature are characterized by a high ethanol content (solvent ratio *S* = [H_2_O]/[EtOH] < 1).

The potential SFME character of the H_2_O/EtOH/TEOS system is usually not considered, and a potential self-templating effect of the ternary system as SFME is poorly investigated.^25^ In other publications, the templating effect of an existing SFME on the synthesis of Stöber particles is investigated. First, Sun et al.^17^ investigated the pseudoternary system water/EtOH/dichloromethane+TEOS in the O/W region and showed smaller particles with smaller distributions compared to those derived from similar ternary mixtures (at equal solvent ratio *S*). Later, Gudelj et al.^23,24^ investigated the systems H_2_O/EtOH/heptanol + TEOS and H_2_O/EtOH/pentanol + TEOS in the pre-Ouzo/bicontinuous (BIC) region and showed the potential use of these pseudoternary systems.

Three aspects should therefore be examined more closely in these systems. How does the classical Stöber process behave at low catalyst concentrations ([TEOS]/[NH_3_] > 1) and high water content (*S* ≥ 1)? How do the different regions/compositions of the SFMEs or the choice of the oil component influence the resulting particles? Does the ternary system of H_2_O/EtOH/TEOS form a SFME with potential self-templating for particle synthesis?

## 2 Experimental Section

### 2.1 Materials

The following chemicals were used as received without further purification: tetraethyl orthosilicate (TEOS, Sigma-Aldrich, ≥98%), ethanol (EtOH, Sigma-Aldrich, ≥99.8%), 25 wt % ammonium hydroxide solution (NH_3_, Supelco, p.A.), toluene (Fisher Scientific, p.A.), and 1-octanol (TCI chemicals, > 99.5%). Ultrapure water from a Millipore system (resistivity >18 MΩ·cm).

### 2.2 Phase Diagram Construction

2 g of the binary mixtures of water/hydrotrope or oil/hydrotrope were prepared in screwable glass tubes to record the ternary phase diagrams. The samples were tempered to 25 °C with the heating thermostat A 111 and the immersion thermostat A 100 from Lauda (Lauda-Königshofen). After the tempering, the third component is added drop by drop with a pasteur pipet outside of the thermostat and mixed thoroughly. The change in phase behavior was determined by the turbidity of the sample with the naked eye. The samples were tempered again to 25 °C. Changing the temperature can cause the system to become single-phase and clear again. For these samples, the process was repeated until the system remained turbid at 25 °C. The samples were weighed to determine the amount of the third component. Weight fractions and molar fractions were then calculated from the masses.

### 2.3 Synthesis of Silica Nanoparticles

Silica nanoparticles were synthesized from points C, f, g, and j of the respective ternary and pseudoternary phase diagrams (cf. Tables S1 and S3) by adding the 25 wt % ammonium hydroxide solution as a catalyst to the premixed H_2_O/EtOH/TEOS or H_2_O/EtOH/(oil + TEOS) system at room temperature. The absolute amounts of each component are given in Table 1. Reaction progress was monitored by the kinetic dynamic light scattering (DLS) measurements described in Section 2.5.

**Table 1:** Weight in the Chart for the Synthesis of the Particles at the Points in the Differing (Pseudo)­Ternary Systems

point	*V* (NH_3_) [mL]	*m* (H_2_O) [g]	*m* (oil + TEOS/TEOS) [g]	*m* (EtOH) [g]
C	0.0200	1.9048	0.2381	2.8571
f	0.0102	0.4910	1.0759	1.7931
g	0.0143	0.6800	1.4731	1.2169
j	0.0083	2.1500	0.8500	2.0000

### 2.4 DLS Measurements

Information about the structuring of the SFME samples and the size of the formed nanoparticles and their aggregates were obtained by dynamic light scattering measurements. To this end, 1 mL of the respective samples were filtered through 0.2 μm PTFE membranes and transferred into dust-free cylindrical borosilicate glass cells of 10 mm outer diameter. The sealed DLS tubes were placed in the temperature-controlled toluene bath of a CGS-3 Compact Goniometer System from ALV, which was equipped with an ALV/LSE-5004 Multiple Tau Digital Correlator and a vertically polarized 22 mW He–Ne laser (λ = 632 nm). Measurements were performed at 25 °C and a scattering angle of 90°. The sampling time for any measurement, except the kinetic studies, was 300 s. From the collected data, the mean intensities of the scattered light and the time-dependent self-correlation functions were determined. Average hydrodynamic diameters of the scattering species were extracted via a second-order cumulant analysis^51^ and the CONTIN algorithm,^52,53^ although we are aware that this procedure may be questionable for ternary or quaternary mixtures. Filtration through 0.2 μm PTFE membranes removes airborne dust and other particulate contamination prior to each measurement. Such contaminants are several orders of magnitude larger than the fluctuating SFME nanostructures and, once present, dominate the scattered intensity. The filter pore size greatly exceeds the reported SFME aggregate sizes, so filtration itself is not expected to alter the measured structures.

### 2.5 Kinetic Measurements

Repeated DLS measurements were taken for the kinetic experiments. To this purpose, 300 s measurements were taken every 15 min for the samples of the ternary mixture H_2_O/EtOH/TEOS until there was no more visible change in the obtained correlation function. For the samples containing oil and with arginine as the catalysts, the same procedure was sufficient. Because of accelerated reaction rates with the added catalyst NH_3_, the measurement was changed to 30 s measurements every 30 s for at least an hour or until visible precipitation occurred.

### 2.6 SWAXS Measurements

Small- and wide-angle X-ray scattering (SWAXS) data were recorded on a Nano-inXider from Xenocs (Grenoble, France), equipped with two detectors. A PILATUS3 R 100 K from Dectris (Baden-Daettwil, Switzerland) with a sample-to-detector distance of 948 mm was used as SAXS detector without a beamstop, in principle covering a *q* range of 0.0057–4.47 nm^–1^, where *q* = 4π·λ^–1^·sin­(θ/2) is the magnitude of the wavevector, and θ is the scattering angle. The transmission was measured simultaneously. Due to the beamstopless setup, the evaluable q range is limited by the direct beam, which influences the measured intensity up to *q* = 0.2 nm^–1^. Another PILATUS3 R 100 K detector from Dectris, centered at an angle of 33° with respect to the direct beam, with a sample-to-detector distance of 80 mm, was used as a WAXS detector, covering a *q* range of 3.47–40.83 nm^–1^. The WAXS detector was protected by a Mylar window, while the entire beam path from source to SAXS detector was windowless. A microfocus sealed tube (Cu, 30 W, 40 μm focus) generated X-rays with a wavelength of 0.154 nm (Cu Kα line). Measurements were performed under vacuum (≈0.1 mbar) in the “High Resolution” configuration of the Nano-inXider with a beam size on the sample of around 400 μm, a typical flux of 15 Mph·s^–1^, and a resolution of Δ*q* = 0.024 nm^–1^. Samples were measured at room temperature (20–25 °C, typically ≈23 °C) in individual capillaries made of borosilicate glass (nominal outer ø 1.5 mm, wall thickness 0.01 mm, length 80 mm) from WJM-Glas/Müller GmbH (Berlin, Germany). Capillaries were sealed using a glue gun. The capillary thickness was measured with a precision of ±0.01 mm at the beam position using a spatial intensity scan perpendicular to the capillary and beam axes, achieved by moving the capillary, i.e., the sample stage, relative to the beam. Samples were measured in single acquisitions with exposure times of 1200 or 10,800 s, and error bars correspond to the statistical error of the measured intensity. One-dimensional (1D) data are radially averaged and normalized to the capillary thickness. Empty capillary scattering was subtracted as background from all sample data. Data were corrected for flat field, spatial distortion, electronic and dark noise, and transmitted flux. The Nano-inXider is regularly calibrated using glassy carbon to obtain an absolute scale, as well as silver behenate and lanthanum hexaboride to ensure correct q-mapping for SAXS and WAXS, respectively. The “absolute” intensity obtained from the Xenocs Nano-inXider software (version 2.3.5) was manually corrected by a factor of 0.909 based on a Lupolen (thickness of 2.38 mm, reference absolute intensity *I*(*q*) = 6.00 cm^–1^ at *q* = 0.37 nm^–1^) reference measurement. The absolute intensity was also confirmed by measuring a water capillary, yielding a constant intensity for water around the reference value of 0.01642 cm^–1^ (at 23 °C). The q-mapping was also checked and corrected by measuring silver behenate in a capillary.

### 2.7 TEM Measurements

To elucidate the nanostructure of the formed particles, 10 μL of the as-obtained dispersions were placed on copper grids (400 mesh × 62 μm pitch) that were coated with a thin layer of graphite (using a Carbon Coater 208carbon from Cressington). After soaking for 20 s, excess liquid was removed by blotting with a filter paper. The fixed specimen on the grid was washed with 20 μL of water and then dried again by blotting. Finally, the grid was left to dry in a closed Petri dish on a filter paper for at least 24 h, before it was transferred into the electron microscope using a Gatan cryo-holder.

Samples were screened and imaged with a 200 kV field-emitter TEM (JEM-2100F, JEOL) and a 16-megapixel complementary metal–oxide–semiconductor camera (F416, TVIPS). Microscope and imaging parameters were controlled using SerialEM software (version 3.8).

## 3 Results and Discussion

### 3.1 Ternary Phase Diagrams and Structuring of Different Mixtures

#### 3.1.1 H_2_O/EtOH/TEOS Phase Diagram and Structuring of Various Points

The phase diagram of the H_2_O/EtOH/TEOS ternary system is shown in weight fraction (cf. Figure 2a) and in molar fractions (cf. Figure 2b). The gray areas in Figure 2 visualize the two-phase region with the single-phase region above, uncolored. The hydrolysis rates of TEOS at neutral pH are slow enough that they can be ignored for the duration of the phase diagram construction (<20 min).^25,54,55^

**Figure 2:**
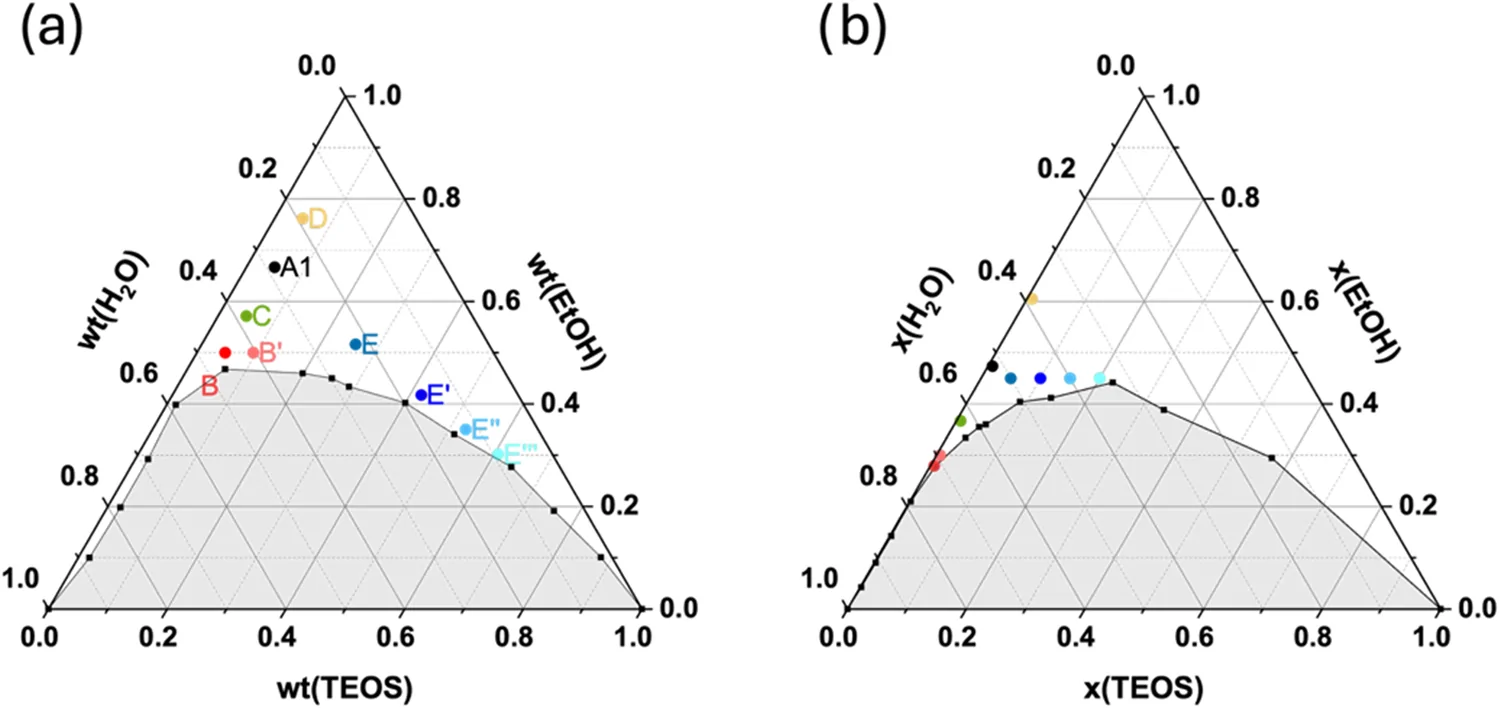
Phase diagrams of the ternary mixture of H_2_O/EtOH/TEOS expressed in weight fraction wt (a) and molar fraction × (b). The exact fractions of the various points visible in parts (a) and (b) are given in Table S1.

Various points are shown in Figure 2a,2b, and their exact composition is given in Table S1. The points A1 to D are located in the part of the phase diagram which is used in most of the publications regarding Stöber silica particle formation (cf. Figure 1) and are chosen in a way to directly compare the differing results to Lu et al.^25^ A series of additional points (E to E″) near the phase boundary were chosen to further characterize the system at higher TEOS concentrations.^12^

The time correlation function, and therefore the structuring, at these points can be seen in Figures 3a and S1, respectively. These are typical correlation functions of ternary mixtures with little to no structuring^56,57^ and strongly oppose the results shown by Lu et al.^25^ with “SFME” aggregate sizes of around 200 nm. Their methodology of crafting the SFME samples, in particular the long stirring time and sonification, suggests that particles were already formed, in an uncontrolled way, before the characterization. Typical SFME aggregate sizes are usually below 100 nm.^58,59^ Further, the DLS is limited to analyze these samples for roughly the aggregate size, as only the composition, but neither the viscosity nor the refractive index of the outer pseudophase is known.

**Figure 3:**
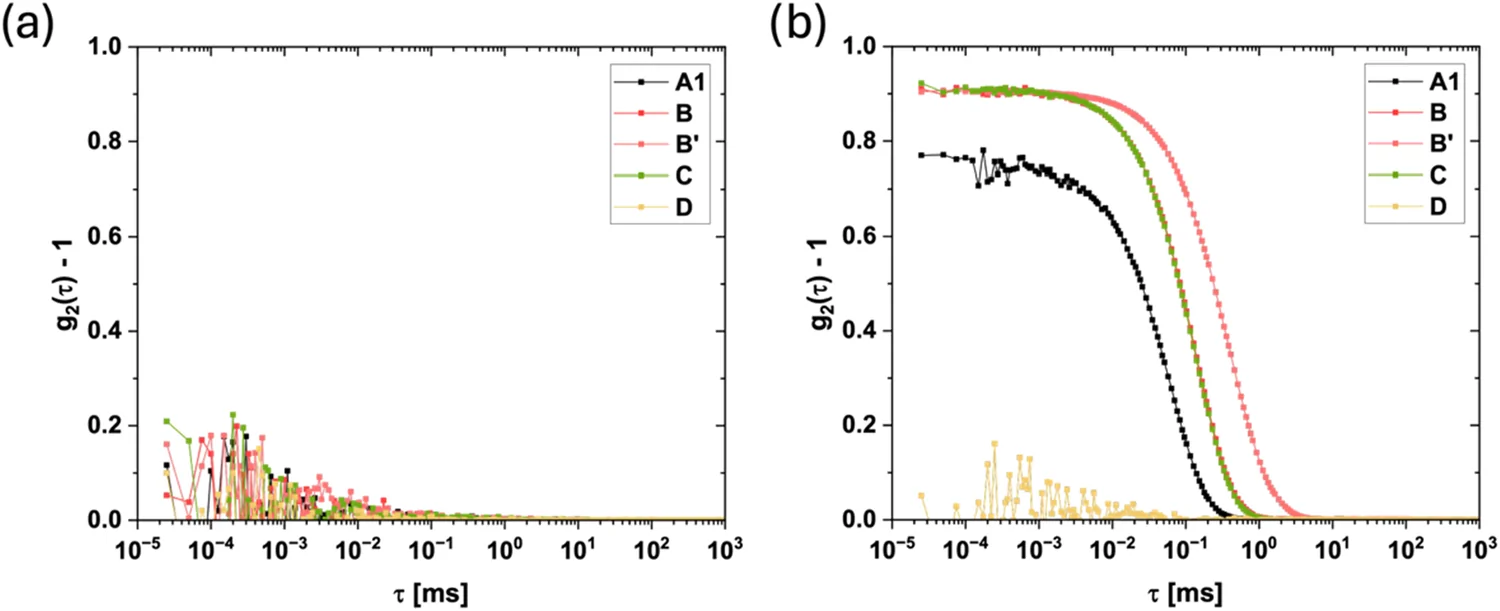
Correlation functions of DLS measurements of the respective points A1, B, B′, C, and D directly after preparation (a) and after 4 days without stirring (b). The exact compositions of the various points shown in parts (a) and (b) are given in Table S1, and the sizes of the formed particles in Table S2.

To substantiate these claims, additional SAXS measurements (cf. Figure S2a) of the points C and B were made. These points are the closest to the pre-Ouzo region and should, based on previous results, therefore be the most structured in Figure 2.^58,59^ Both methods combined show that little to no structuring occurs in this system, indicating that instead of SFME formation, the system remains a molecular solution in the observed single-phase region.

Also visible in Figure 3b are the same samples as shown in Figure 3a after a standing time of 4 days. In four of the five compositions, a substantial number of particles is formed after the waiting time shown by the unambiguous correlation function. The evaluated size, using cumulant analysis,^51,60^ is shown in Table S2. Even after 4 days, every sample shows significantly smaller particle sizes and narrower size distributions than what Lu et al. claim to be the aggregates of the SFME (cf. Table S2).^25^ After further standing time, all samples transformed into gels.

Nevertheless, typical trends for Stöber systems are visible as follows:

The lower the water content, and therefore the catalyst (for the hydrolysis reaction) concentration, the slower is the reaction rate and the smaller are the formed particles (D < A1 < C < B).^3,5^

The higher the TEOS content, the bigger are the resulting particles (B′ > B).^61,62^

Due to the neutral pH value, there is no uniform nucleation with subsequent growth phase but rather an extended growth phase across the entire volume until a uniform gel is formed.^3^

These results clarify that the H_2_O/EtOH/TEOS ternary system does not form a SFME. This does not disqualify the system near the phase boundary for Stöber synthesis in general but gives first hints that the mechanism proposed by Lu et al. is incorrect.^25^ SFME aggregates do not influence the size of formed particles in this ternary system, as there are no structures found in the solutions. The interest in the synthesis with high water concentrations still remains unchanged, since limited experiments have been conducted in that area of the ternary phase diagram (cf. Figure 1).^32,41^

#### 3.1.2 H_2_O/EtOH/(Oil + TEOS) Phase Diagram and Structuring of Various Points

To actually evaluate the influence of SFME aggregates on Stöber particle synthesis, an existing SFME is used, and 10 vol% of TEOS are added to the oil prior to SFME formation. In comparable studies, the TEOS is added after the SFME formation, without considering the non-negligible shift of the point in the ternary phase diagram nor the potential shift of the phase boundary.^17,23,24^ To evaluate the actual positions of points used for Stöber synthesis, pseudoternary phase diagrams of the systems H_2_O/EtOH/toluene + TEOS (red) and H_2_O/EtOH/1-octanol + TEOS (green) were constructed (cf. Figure 4).

**Figure 4:**
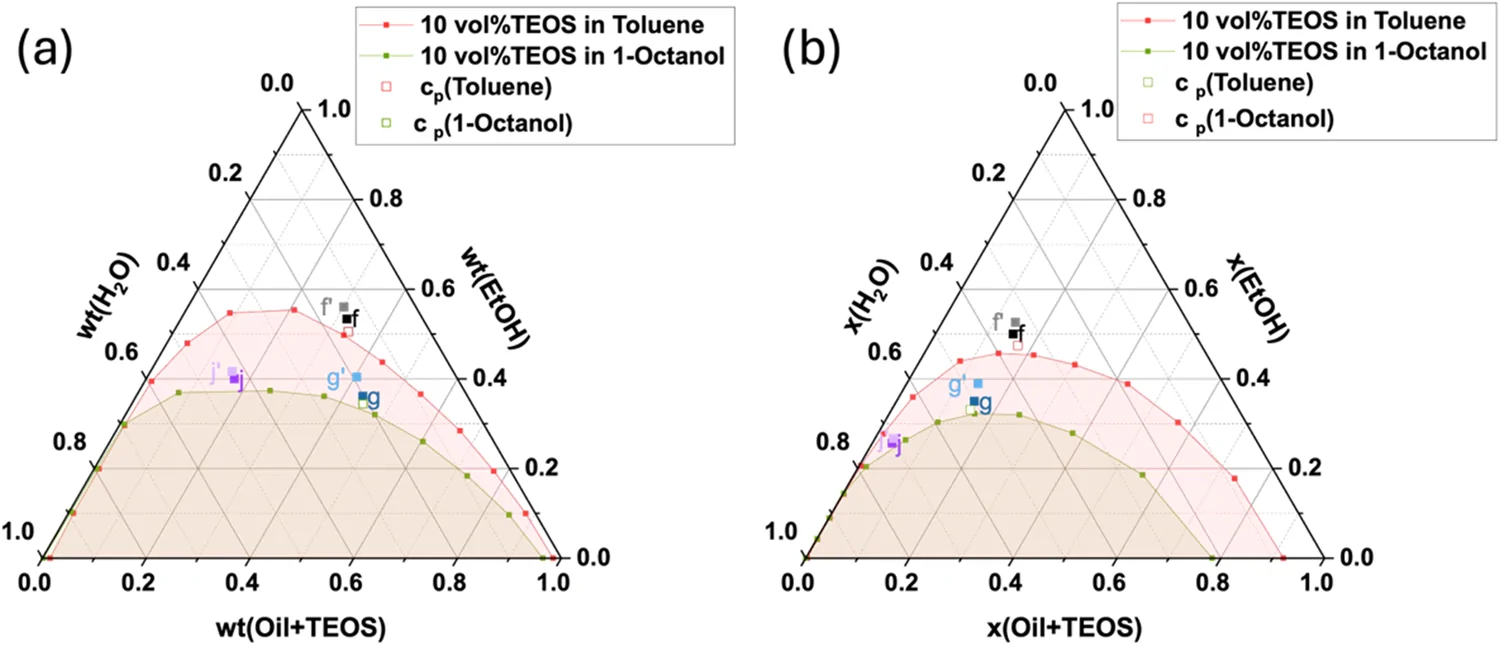
Phase diagrams of the ternary mixture of H_2_O/EtOH/Oil + TEOS expressed in weight fraction wt (a) and molar fraction × (b). The red phase boundary represents 10 vol% TEOS in toluene, and the green phase boundary represents 10 vol% TEOS in 1-octanol. The exact fractions of the points f, j, g, f′, j′, and g′ visible in parts (a) and (b) are shown in Table S3. The points f′, g′, and j′ correspond to their counterparts with a theoretical shift after complete TEOS hydrolysis. The critical points of the ternary mixtures are indicated by the respective colored squares.^63,64^

Again, both the ternary phase diagrams are shown in weight fractions (cf. Figure 4a) and in molar fractions (cf. Figure 4b). Compared to the ternary phase diagrams of pure toluene^64^/1-octanol,^65,66^ the biphasic area of the phase diagram decreases slightly (cf. Figure 4). The points f, g, and j and their respective counterparts f’, g’, and j’ (theoretical shift after complete TEOS hydrolysis) are visualized in both ternary phase diagrams, and their exact compositions are given in Table S3.

Point g was chosen because of its proximity to the phase boundary as well as the Ouzo region (close to the critical point), which results in the highest amount of pre-Ouzo aggregates.^58,59^ The H_2_O/EtOH/toluene system shows the most pronounced structuring around its critical point in the oil-rich region.^66^ Since this region is not of interest here, and only very weak structuring is indicated in the water-rich region, f was chosen at a point with relatively high water content, where structuring is still significant. g was chosen at the same oil+TEOS molar fraction as f with 1-octanol instead of toluene.

f′, g′, and j′ represent the theoretical shift of the respective points f, g, and j after complete hydrolysis of TEOS (1 TEOS releases 4 EtOH). DLS and SWAXS measurements of these points are visualized in Figure 5a,5b, respectively.

**Figure 5:**
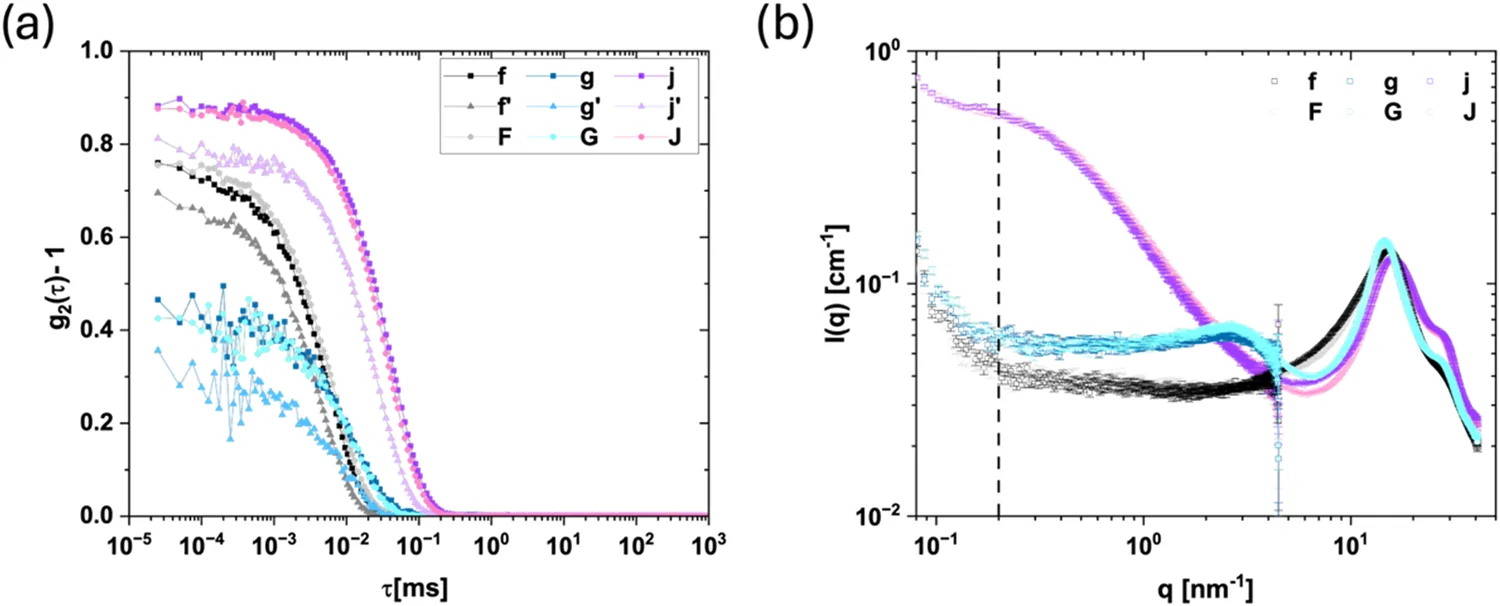
(a) Correlation function of DLS measurements of the points f, g, and j and their respective alternated points after a theoretical complete TEOS hydrolysis f′, g′, and j′. The points F, G, and J represent the same points without the addition of TEOS. Samples were prepared immediately before the measurement. (b) SWAXS spectra of the points f, F, g, G, j, and J. The increase at *q* < 0.2 nm^–1^ results from the X-ray beam (no beamstop).

The increase of the EtOH concentration through complete hydrolysis of the small amount of dissolved TEOS (10 vol% in oil) already results in a visible decrease in the correlation in all systems (cf. Figure 5a). This indicates a reduction in the amount of present scatterers and also hints, in combination with decreased decay times (τ), at reduced aggregate sizes. The hydrodynamic radius (*r*_H_) could be evaluated, as the composition of the pseudophases can be estimated from the SAXS measurements (cf. Figure 5b, Table S4).^57,58^ However, the usefulness of such information is questionable when considering the dynamics of the aggregates in an SFME system. Instead, the lag times (τ) derived from DLS contain both information about size and the fluctuation rate of the aggregates.^8,58,67^ Therefore, the aggregates in j persist longer than in g and f, the latter having the highest dynamics and thus the shortest aggregates lifetime. Additional SWAXS spectra (cf. Figure 5b) confirm that the incorporation of 10 vol% TEOS in the oil-rich pseudophase does not change the formed structures significantly for either sample. While for the sample j, direct pre-Ouzo aggregates are clearly visible in both the DLS measurement and SWAXS spectra, the sample f shows ambiguous results. The missing indication for aggregates in the SWAXS spectra possibly results from the poor contrast between the pseudophases in this system (cf. Figure S2b), while the DLS measurement approves the existence of aggregates. The SWAXS spectra for g clearly indicate a position in the BIC area of the ternary system, while the DLS measurement only confirms the existence of some kind of aggregates (cf. Figure 5).^12,58^

### 3.2 Kinetics of Stöber Nanoparticle Formation in the Different Mixtures (Refutation of Mechanisms Proposed in Literature)

#### 3.2.1 Kinetics of Point C (H_2_O/EtOH/TEOS)

Point C was chosen for further investigation, as only a few results with similar high water contents are reported in the literature (cf. Figure 1).^32,41^ Time-dependent DLS measurements were conducted without catalyst (cf. Figure S3a), with arginine (cf. Figure SII-10 inspired by ref 68) as catalyst, and with NH_3_ as catalyst (cf. Figure 6a). In all cases, with and without a catalyst, a change in the correlation functions is visible: From no scattering in the beginning (yellow) to a clearly defined correlation (purple) after different times, depending on the system.

**Figure 6:**
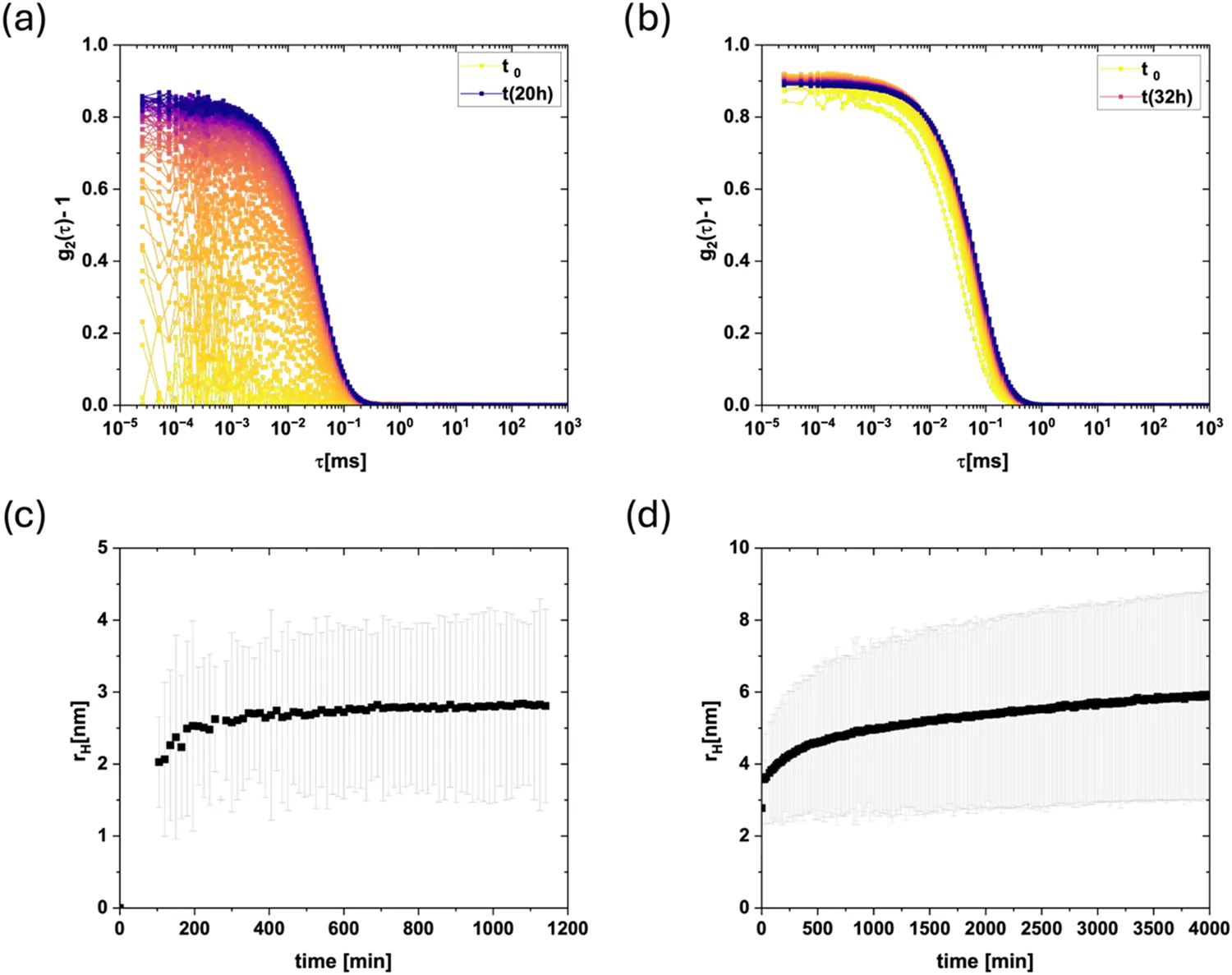
Correlation function of multiple DLS measurements during the formation of particles in the ternary mixture H_2_O/EtOH/TEOS at point C with the addition of NH_3_ as catalyst (a) and a regrowth step after 24 h (b). The gradient from yellow (*t*_0_) to purple (*t*_*e*nd_) visualizes the change of the samples over time. The corresponding change/increase in hydrodynamic radius (*r*_H_) derived from time-dependent DLS measurements is visualized in parts (c) and (d), respectively. The hydrolysis ratio is *R* = 92.5, and the solvent ratio is S = 1.7.

The addition of NH_3_ as a catalyst not only reduces the reaction time but also induces a size restriction for the formed particles (cf. Figures 6a and S3a). Instead of infinite growth to form a uniform gel, particles with a capped size are formed (cf. Figures S5a and 6c).^3^ Contrary to the mechanism proposed by Lu et al.,^25^ no SFME aggregates are present in the solution that can then be turned into particles of similar size or even coagulate into larger aggregates to form even larger particles. Instead, typical Stöber-like particle formation is observed. The used NH_3_ concentration ([TEOS]/[ NH_3_] = 8.9) is significantly lower compared to particles synthesized in literature at comparable ternary mixtures ([TEOS]/[ NH_3_] = 0.6 ^41^ or 0.003 ^32^), resulting in higher reaction times (∼20 h vs 1 h). These slower reaction times generate further possibilities to control the size of the formed particles.

The addition of further TEOS, after equilibrium has been reached (approximately 24 h, cf. Figure 6c), results in uniform growth of the particles. Therefore, a visible shift of the correlation function to higher lag times (τ) is visible without the emergence of a second correlation indicating growth instead of the formation of new particles (cf. Figure 6b). After another 24 h, the particles can be doubled in size without any change of further parameters.

Even though the system does not form SFME aggregates, the investigation of particles formed at high water content (S > 1) and low catalyst concentrations ([TEOS]/[NH_3_] > 1) appears interesting, especially given the limited data available in the literature.^32,41^

#### 3.2.2 Kinetics in Regular SFME Systems

After the addition of the catalyst NH_3_, changes in all samples f, g, and j are visible. After some time (>1 h), a white precipitate is visible for f and g, while j remains as a clear solution even after 3 days. The [TEOS]/[NH_3_] ratio equals 8.9, as before for point C, for all of the experiments. The hydrolysis ratios for g and f are similar at *R* = 46, but the solvent ratios are rather different at *S* = 1.4 and *S* = 0.7, respectively. j shows a significantly higher hydrolysis ratio of *R* = 252 and a solvent ratio of *S* = 2.7. Time-dependent DLS measurements were conducted after the addition of NH_3_ to evaluate particle growth (cf. Figure 7).

**Figure 7:**
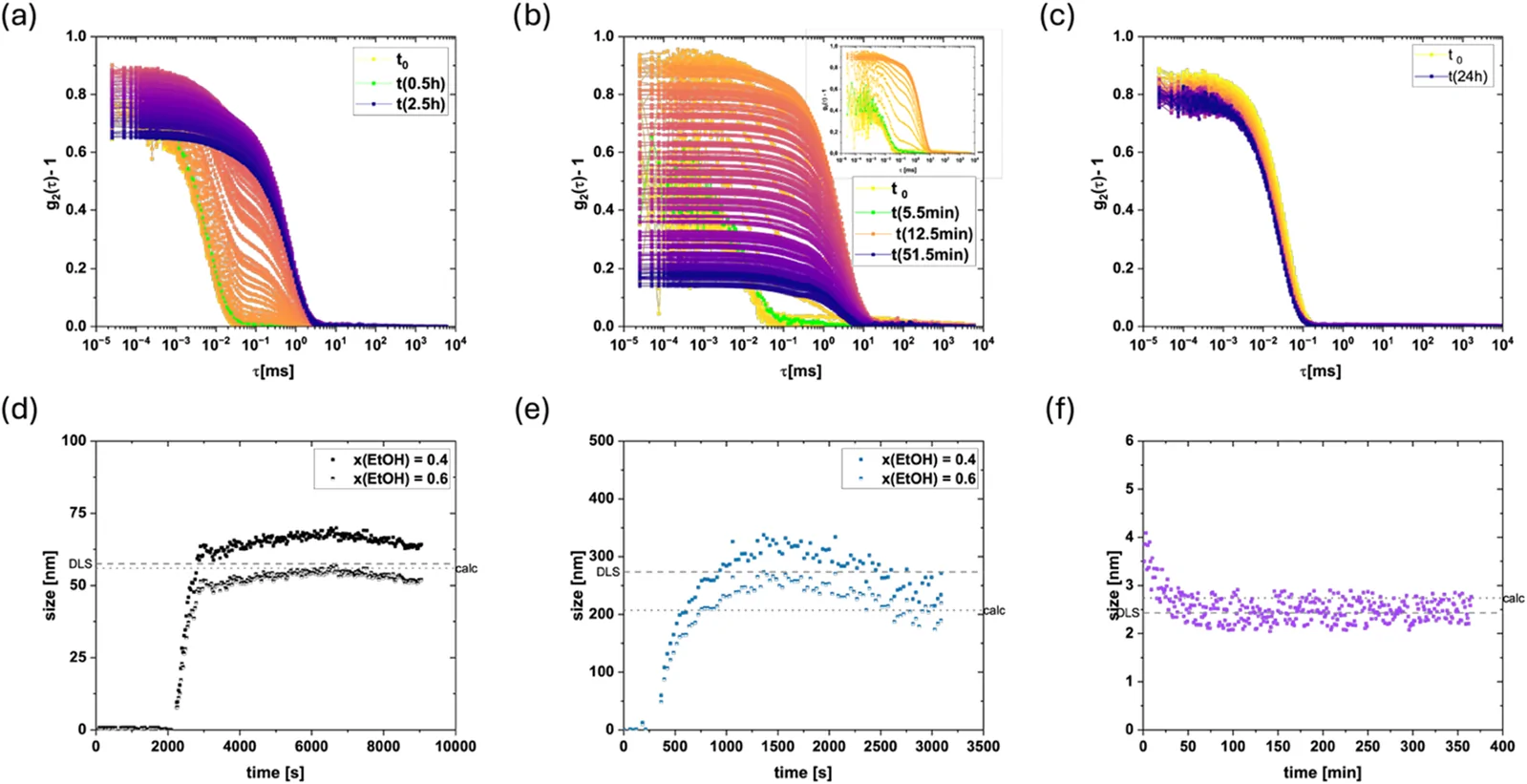
Correlation function of multiple DLS measurements during the formation of particles after the addition of NH_3_ to (a) f, (b) g, and (c) j. The gradient from yellow (t0) to purple (tend) visualizes the change of the samples over time. The corresponding change/increase in hydrodynamic radius (r_H_) derived from time-dependent DLS measurements is visualized in parts (d), (e), and (f), respectively. The dashed (DLS) and the dotted (calculated) lines correspond to the values shown in Table S5. As the exact distribution of ethanol changes as the reaction progresses, only the boundaries of the range, within which the *x*(EtOH) is most likely, were plotted [TEOS]/[NH_3_] = 8.9.

The three shown points exhibit vastly different reaction kinetics. The first change of the correlation function for sample f (cf. Figure 7a) is visible after 30 min, and a second fraction of scatterers emerges at τ values 2 orders of magnitude greater. While the correlation function of sample g (cf. Figure 7b) shows a similar trend after 5 min, the sedimentation is faster, resulting in almost complete sedimentation after 50 min. In contrast, the correlation function of sample j (cf. Figure 7c) remains almost the same for over 24 h and appears to shift toward smaller lag times (τ).

All samples share that the scattering of the SFME structures/the fluctuations in these measurements remain visible. Instead of a shift in the correlation function, which would be expected with growing structures/aggregates, a second fraction of scattering centers appears after some time (f and g). The SFME structure remains visible until the number of large scattering centers becomes too large, as the scattering intensity is strongly size-dependent (∼*r*_H_^6^).^69^

The change in sample j indicates that the second fraction of scatterers that emerges must be “smaller” than the initial SFME aggregates, but less than one order of magnitude, which would lead to a shift of the correlation function (cf. Figure 7c).

Size evaluations of these measurements are found in Figure 7d–f and Table S5. After the initial delay, the evaluated size remains almost constant for an extended period of time for all samples derived from the BIC area. This initial induction period is not observed for the classical Stöber process but was seen before in synthesis in an O/W SFME system^17^ and is typical of synthesis in microemulsions.^70,71^ In analogy to Sun et al.,^17^ the induction period was followed by a nucleation burst with rapid growth and a slow increase stage (cf. Figure 7). For f and g, the size stagnation stems from uniform growth and the onset of sedimentation, which appears to maintain the size balance of the dispersed particles. For j, the particles are so small that they neither aggregate nor sediment.

The difference between g and j, which are compositions of the same ternary system, is most likely due to the different SFME structures present. In j, pre-Ouzo aggregates are clearly prominent and therefore a high fraction of interphase is present, increasing the diffusion of the TEOS into the pseudowater phase. Sample g is in the BIC area, which has less interphase, resulting in reduced TEOS diffusion into the pseudowater phase. Also, the higher hydrolysis ratio in j (252 vs 46) increases the rate of hydrolysis, especially at the start of the reaction, resulting in more nuclei and, in turn, smaller particles.

### 3.3 Characterization/Comparison of Formed Stöber Nanoparticles

To evaluate the size and morphology of the formed particles after a one hour reaction time, TEM micrographs were taken (cf. Figure 8). TEOS-to-silica conversion for these four systems was estimated from literature data for comparable ammonia-catalyzed Stöber-type syntheses rather than measured directly. Elemental analysis of dried Stöber silica reported approximately 92.7 wt % SiO_2_, with the remainder attributable to residual ethoxy groups and adsorbed water.^30^ This is consistent with the standard practice of assuming complete TEOS-to-silica conversion when calculating theoretical silica volume fractions^72^ and is further supported by the direct, time-resolved tracking of the conversion of soluble Si species into insoluble SiO_2_ via a ^29^Si NMR mass balance, cross-validated by SAXS and DLS.^42^ The range, 92.7% to the theoretical maximum of 100%, was used together with the measured size (cf. Table S5) to calculate the resulting silica mass and particle number listed in Table S6.

**Figure 8:**
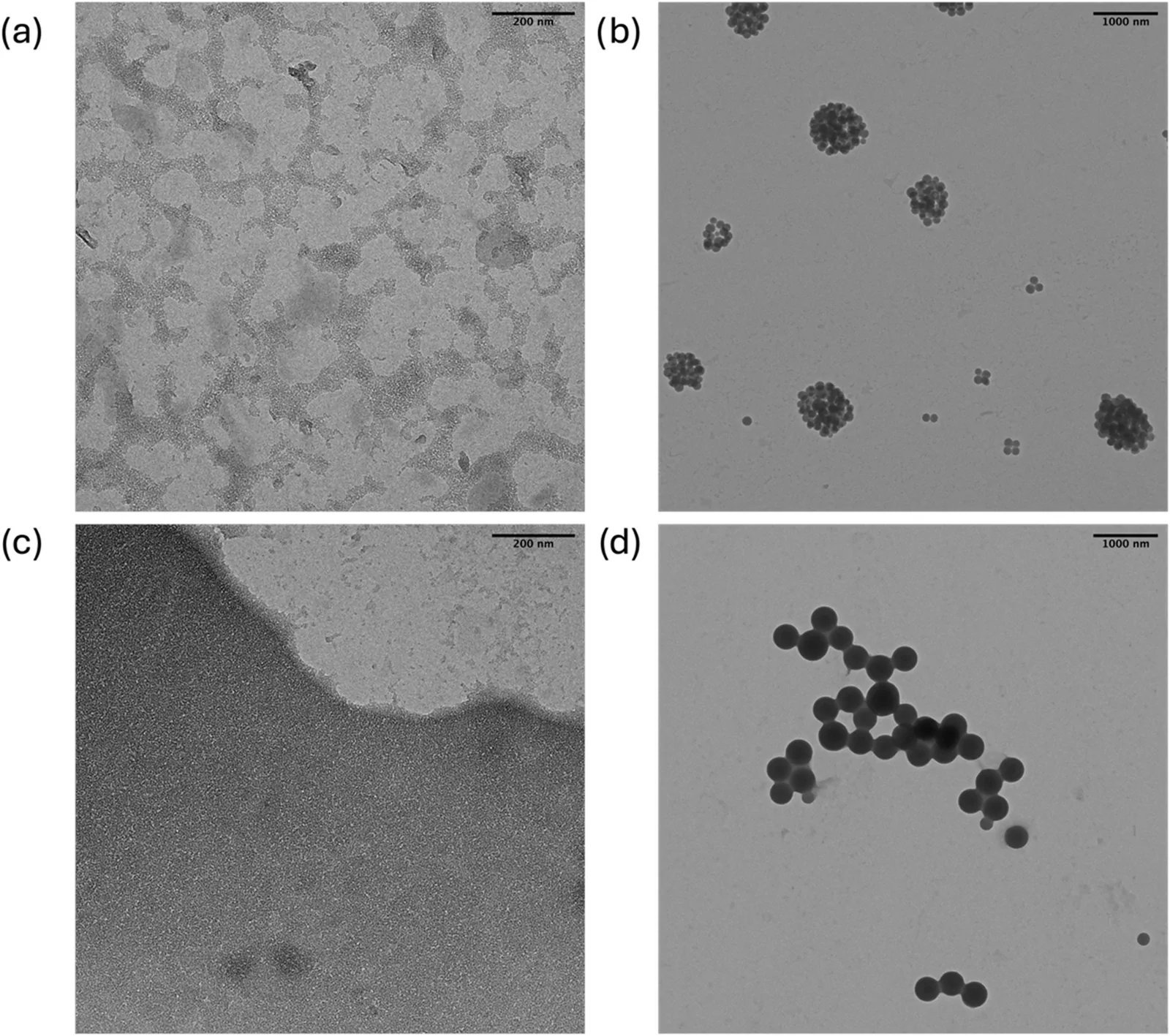
(a) TEM micrograph of silica particles derived from point C in the H_2_O/EtOH/TEOS system at a magnification of 40000x. The scale bar corresponds to 200 nm. (b) TEM micrograph of silica particles derived from point f in the H_2_O/EtOH/toluene + TEOS system at a magnification of 6000x. The scale bar corresponds to 1000 nm. (c) TEM micrograph of silica particles derived from point j in the H_2_O/EtOH/octanol + TEOS system at a magnification of 40000x. The scale bar corresponds to 200 nm. (d) TEM micrograph of silica particles derived from point g in the H_2_O/EtOH/octanol + TEOS system at a magnification of 6000x. The scale bar corresponds to 1000 nm.

The particles derived from sample C (cf. Figure 8a) are highly aggregated on the TEM grid but clearly visible. Information about the particle size can be derived from measurements of the FFT pictures (cf. Figure S6a) of the shown TEM micrograph. The visible particles are between 5 and 13 nm in size (cf. Table S5) and therefore continue the trend of reduced particle size for low NH_3_ concentrations at high hydrolysis ratios *R*, as shown in the literature.^32,41^

The particles derived from sample j (cf. Figure 8c) are quite similar. They are also heavily aggregated on the grid and size evaluation by measurements of the FFT (cf. Figure S6b) is comparable, suggesting slightly smaller aggregates. These particles are between 3 and 8 nm (cf. Table S5).

The particles derived from sample f (cf. Figure 8b) and sample g (cf. Figure 8d) show rather different results. The formed particles are significantly larger with ∼58 and ∼207 nm, respectively (cf. Table S5). They are clearly spherical with sharp edges and are slightly aggregated on the TEM grid. Such clustering is not indicative of templating by discrete oil droplets, since the SFME does not contain stable droplets of this size to begin with. Instead, it most likely results from a combination of ongoing particle aggregation and fusion, a process well documented for Stöber-type silica over time, and from sample drying on the TEM grid during preparation.^36^

The similarity between C and j indicates a similar particle formation process, even though j shows SFME (pre-Ouzo) aggregates, while C does not. The hydrolysis ratio of j is almost three times higher than that for C, and the solvent ratio is almost doubled. This results in faster hydrolysis/condensation kinetics, which in turn increases the number of nuclei capping the growth of the particles at a smaller final size.^5,31^

Also, the overall TEOS concentration in j is halved, reducing the size due to a shorter growth phase. The point j significantly decreases the size and size distribution, hinting at no direct templating effect by pre-Ouzo aggregates compared to the system used in C.

In the other systems, the formation of the SFME beforehand seems to alter the resulting particles significantly. At the same hydrolysis ratio (∼46), the particles derived from g are almost 4 times bigger than the particles derived from f (cf. Table S5). These size differences can not only be attributed to the solvent ratio or the hydrolysis ratio but also to the influence of the oil component. Compared to SFME systems with primary alcohols,^23,24^ an interesting trend occurs, inverse to the trend visible in classical Stöber syntheses.^38,42,62^ The particle size seems to decrease with increased viscosity (chain length) (cf. Figure S8) of the alcohol (pentanol^23^ ∼ 1000 nm > heptanol^24^ ∼ 700 nm > octanol ∼ 500 nm) at similar points in the ternary system. (Note that such comparisons are to be taken with a grain of salt due to the inconsistencies and no given values for *R*, *S*, or [NH_3_].) Nevertheless, toluene (no primary alcohol, no hydrogen bonding) does not fit into this trend and instead results in smaller particles despite/due to its lower viscosity. It appears that the solubility of the hydrolyzed TEOS species and the solubility of the catalyst (cf. Figure SII-10) in the pseudo-oil phase and the interphase play a decisive role.

Beyond particle size, Table S5 also reports the polydispersity index (PDI) for all four systems, both from DLS cumulant analysis and calculated from the TEM-based size distributions. Point j shows an unambiguously low PDI (0.062 from DLS, 0.032 calculated), among the most monodisperse Stöber-type silica dispersions reported in the literature.^40^ Points C and f fall within, or better than, typical literature ranges for Stöber-type silica.^40^ For g, the DLS-derived PDI (0.224) is markedly higher than, and inconsistent with, the calculated value (0.008); we attribute this to the onset of sedimentation of these comparatively large (∼270 nm) particles during the DLS measurement, which biases the apparent size and distribution width upward rather than reflecting genuine polydispersity.

### 3.4 Proposed Mechanism of Nanoparticle Formation in the Regular SFME Systems

For both the pre-Ouzo region and the BIC region in the ternary phase diagram, a new mechanism can be proposed. Differing to the existing mechanism,^17^ which describes particle formation in the O/W region, the condensation of TEOS most likely happens in the pseudowater phase instead of the pseudo-oil phase. Part of the hydrolysis may happen in the pseudo-oil phase/interphase, depending on the polarity, but the charged hydrolyzed TEOS monomers tend to diffuse into the pseudowater phase (cf. Figure 9). Based on Fick’s First Law,^73^ the molar flux *J* across the interphase can be given by

1 $$J=\frac{\Delta c}{\int_{0}^{\delta }\frac{1}{D\left(x\right)}dx}$$

with the concentration difference between inner and outer pseudophase Δ*c*, the thickness of the interphase δ, and the diffusion coefficient function *D*(*x*), depending on the variation of the viscosity across the interphase. As the diffusion rate depends on the viscosity of the pseudo-oil phase and of the interphase, the same applies to the reaction rates. The bigger the volume fraction of the interphase, which depends on the position in the ternary system (Pre-Ouzo > BIC), the faster is the diffusion across the interphase. While the volume fraction of the interphase is the major factor in the size difference of g and j, the viscosity is the main factor for the difference between g and f. As the viscosity of the pseudo-oil phase and the interphase is much higher for g (cf. Figure S8), the diffusion is strongly hindered, inducing less nuclei at the start of the reaction, resulting in more extensive growth afterward.

**Figure 9:**
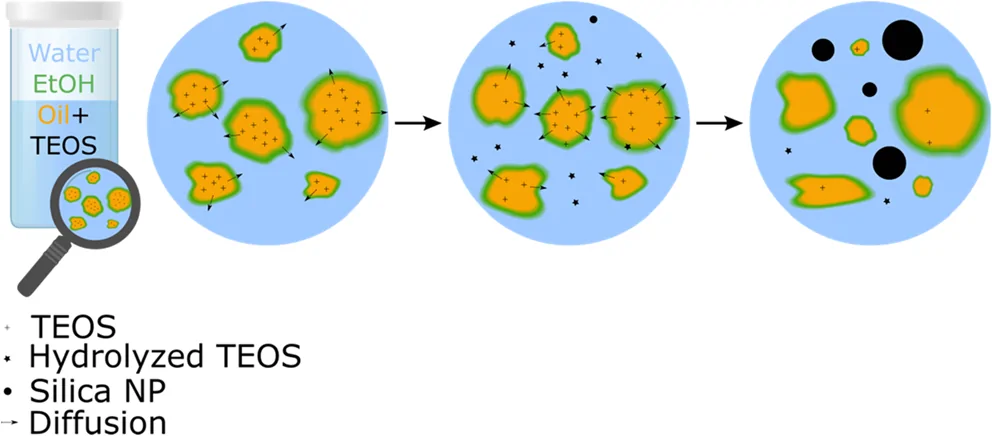
Proposed mechanism of Stöber particle formation in SFMEs.

Additionally, the reaction rate of TEOS hydrolysis is likely reduced in the toluene system. Ammonia is only sparingly soluble in toluene,^74^ so the local coexistence of TEOS and NH_3_ in the interphase is decreased, and mass transfer may become rate-determining. 1-Octanol, as a hydrogen-bond-accepting solvent, dissolves polar solutes such as amines considerably better than toluene does,^75^ which is consistent with the higher observed reaction rate in this system. General treatments of TEOS/silica-species hydrolysis and condensation in polar versus nonpolar media support this picture.^76,77^

Also, the dynamics of the aggregates plays a crucial role, as the rate of forming and dissolving the SFME aggregates influences the contact time/probability of contact between TEOS and the catalyst. This is mainly influenced by the position in the ternary system/closeness to the critical point and the difference in the polarity of water and the used oil. Qualitatively, f shows the fastest fluctuations, followed by g and then by j (cf. Figure 5a), even though the smallest particles were produced there. This indicates that the effect of diffusion on the size of the final particles overshadows the effect of the fluctuation rates.

The mechanism proposed here is best understood as a plausible working hypothesis, rather than an established pathway. Note that the solubility of TEOS and its hydrolysis intermediates in the pseudo-oil phase and the interphase is, at this stage, inferred from the general solvent properties discussed above rather than measured directly for this system. An attempt to localize TEOS within the SFME using a polarity-sensitive dye did not yield a conclusive result, and a more targeted experimental approach remains an open question for future work.

## 4 Conclusion

The ternary system H_2_O/EtOH/TEOS does not form aggregates of a surfactant-free microemulsion. Instead, it is a molecularly dispersed solution throughout the ternary space. Stöber particle syntheses in such systems, characterized by high water content and low [NH_3_], still leave room for research. The formed particles are in the range of few nanometers and, in particular, for point j (PDI = 0.032), exhibit high monodispersity. The high water content enhances the environmental friendliness compared to classical Stöber systems. The addition of regrowth steps allows easy size adjustment.

The incorporation of TEOS into existing SFME systems shows little change to the ternary phase diagrams and the aggregates contained. Depending on the microregion, the SFME structure can exhibit different effects on the synthesized particles. It was evident that particles derived from the BIC area grew in three stages. First, the induction period for nucleation, followed by a nucleation burst and rapid growth, and finally subsequent slow growth.^17^

Also, the chosen oil and, in particular, its viscosity and polarity play an important role in affecting the size, since the TEOS diffusion across the interphase was identified to be the rate-determining step.

SFME structures can induce interesting effects on the synthesis of Stöber particles. While the addition of a hydrophobic compound reduces the environmental friendliness of the system, a new parameter to influence the particle size, shape, and polydispersity is added. At similar *R* and *S* values, two oils, i.e., toluene and 1-octanol, already exhibited significant size differences. Therefore, a study comparing syntheses in several different SFME-forming ternary systems at similar *R* and *S* values would be interesting.

## Supplementary Material



## Notes

Ternary compositions of investigated points in weight and molar fractions (Tables S1, S3, S4); DLS correlation functions and derived hydrodynamic radii of points B–B″, E–E″, and f/g/j (Figures S1, S3–S5, Tables S2, S5); SWAXS spectra of points B and C (Figure S2); FFT analysis and particle-size histograms of TEM micrographs (Figures S6, S7); estimated TEOS-to-silica conversion, silica mass, and particle number (Table S6); viscosities of binary ethanol/solvent mixtures (Figure S8); and literature-derived ternary phase diagrams of Stöber syntheses in molar fractions (Figures S9, S10) (PDF)
