# Supplementary material for: Surfactant-Free Microemulsion as Template for Stöber Silica Nanoparticle Synthesis
Source: ACS Omega. 2026 Oct 13;11(42):65735–46. doi: 10.1021/acsomega.6c08994 (PMC13718209; doi:10.1021/acsomega.6c08994)
Supplement: Supplementary file 1 [file ao6c08994_si_001.pdf]

Supporting Information - Surfactant-free Microemulsion as Template for Stöber Silica Nanoparticle Synthesis

Bastian Rödig, Lena Schmauser, Patrick Denk, Werner Kunz*

Institut für physikalische und theoretische Chemie, Universität Regensburg, D-93053 Regensburg

Table S1. Weight fraction and molar fraction of various points of the ternary mixture H_2O / EtOH / TEOS as shown in Figure 2a, Figure 3, and Figure S1.

	wt(H_2O)	wt(EtOH)	wt(TEOS)	x(H_2O)	x(EtOH)	x(TEOS)
A1	0.286	0.667	0.048	0.519	0.474	0.007
B	0.476	0.476	0.048	0.714	0.279	0.006
B'	0.452	0.500	0.048	0.694	0.300	0.006
B''	0.405	0.500	0.095	0.665	0.321	0.014
B'''	0.357	0.500	0.143	0.632	0.346	0.022
B''''	0.310	0.500	0.190	0.593	0.375	0.032
C	0.381	0.571	0.048	0.626	0.367	0.007
D	0.190	0.762	0.048	0.387	0.605	0.008
E	0.224	0.516	0.259	0.500	0.450	0.050
E'	0.163	0.417	0.419	0.450	0.450	0.100
E''	0.122	0.350	0.528	0.400	0.450	0.150
E'''	0.092	0.302	0.606	0.350	0.450	0.200

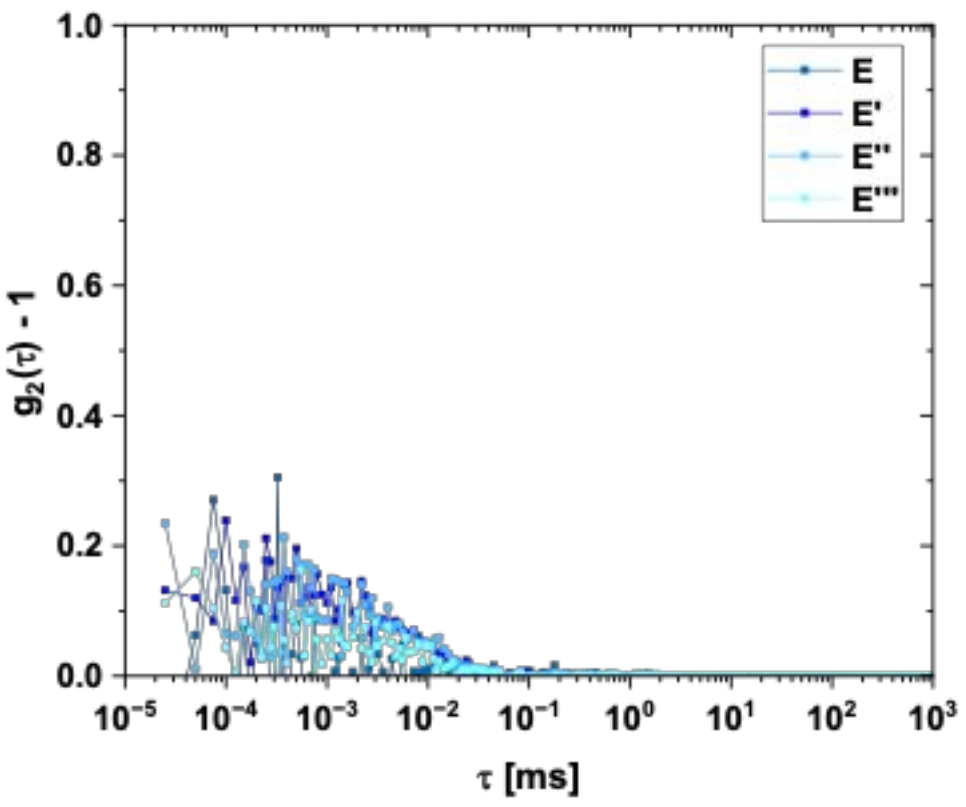

Figure S1. Correlation functions of DLS-measurements of the respective points E, E‘, E’’, and E’’’ directly after preparation. The exact fractions of the various are shown in Table S-1.

Table S2. Hydrodynamic radius (r_H) derived from the DLS measurements shown in Figure 3b. These sizes correspond to particles/aggregates formed after 4d in the respective solutions without catalyst/stirring. For

evaluation the viscosity and refractive index of the corresponding water/ethanol mixture (x(EtOH) = 0.477; 0.281; 0.302; 0.370; 0.610) was used. As the composition D formed no clear correlation function, fitting was not possible.

	r_H [nm]	width [nm]	PD.I.
A1	4.83	2.23	0.213
B	9.37	4.54	0.235
B'	26.67	16.68	0.391
C	9.57	4.55	0.226
D	-	-	-

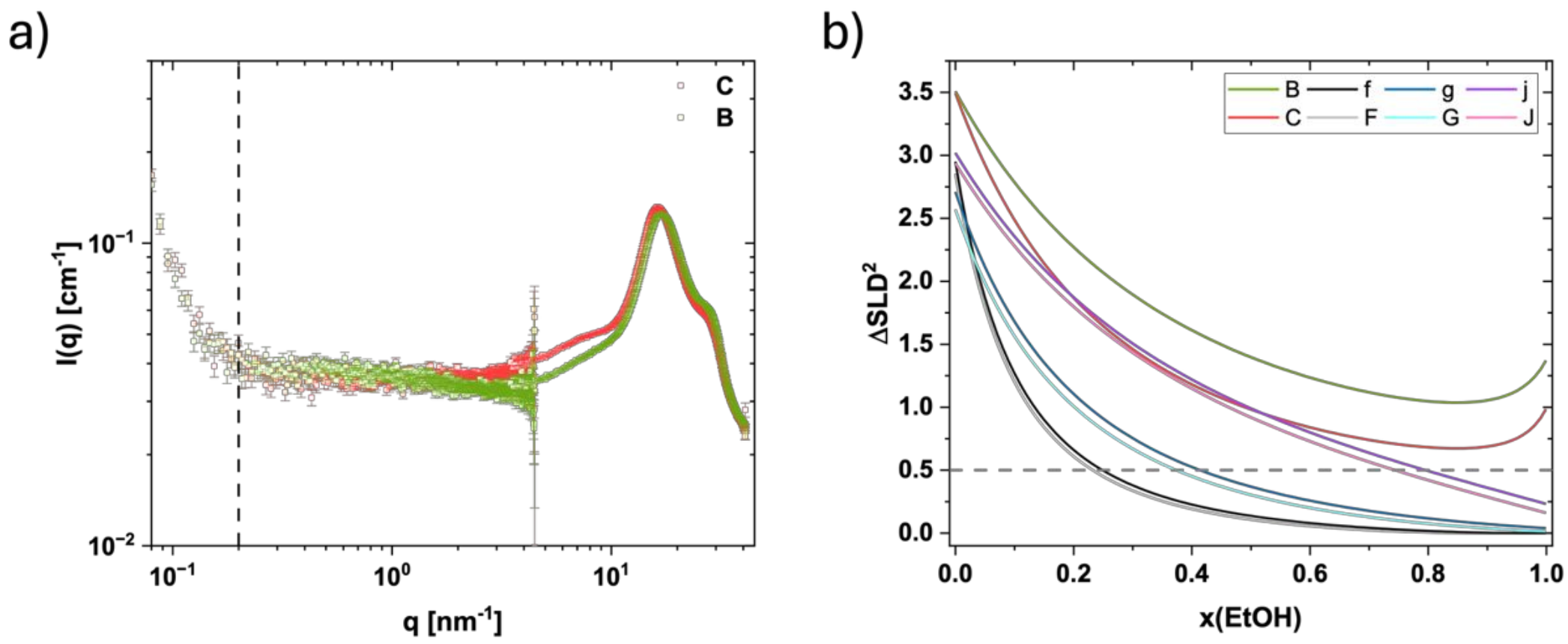

Figure S2. (a) SWAXS spectra of a) point B and C in the H_2O/EtOH/TEOS system. The increase at q < 0.2 nm^{-1} results from the X-ray beam. (b) Visualization of the contrast (ΔSLD^2) in the SWAXS measurements relative to the distribution of the hydrotrope ethanol in the pseudo phases. The molar fraction x(EtOH) indicates the amount of ethanol in the aqueous pseudo phase. In this simplified assumption no oil is solubilized in the aqueous pseudo phase and no water in the oil pseudo phase. The dashed line at ΔSLD^2 = 0.5 shows the threshold below which the resolution of the SWAXS measurements is questionable. For J/j a value of literature was found to be x(EtOH) = 0.6.

Table S3. Weight fraction and molar fraction of various points of the ternary mixture H_2O / EtOH / Oil + TEOS. The point f represents the mixture containing toluene and f' the total hydrolyzed state of the same point. For the points g and j the same is true in the mixtures containing 1-octanol. [1]

	wt(H_2O)	wt(EtOH)	wt(Oil+TEOS)	x(H_2O)	x(EtOH)	x(Oil+TEOS)
f	0.146	0.534	0.320	0.350	0.500	0.150
f'	0.139	0.560	0.301	0.332	0.526	0.141
g	0.202	0.361	0.437	0.500	0.350	0.150
g'	0.192	0.404	0.404	0.473	0.389	0.138
j	0.430	0.400	0.170	0.705	0.256	0.039
j'	0.426	0.417	0.157	0.698	0.267	0.036

Table S4. Estimated x(EtOH) for the points f, g, and j from SWAXS-measurements and their respective hydrodynamic radius evaluated from the DLS measurements. The value for j was taken from literature [1] whereas the values for f and j were estimated to be around 0.4 due to their contrast (Figure S2b) and their position near the critical point.

sample	x(EtOH)	r_H [nm]	width [nm]	PD.I.
f	0.4	0.45	0.17	0.151
g	0.4	0.81	0.38	0.221
j	0.6	3.73	0.59	0.0247

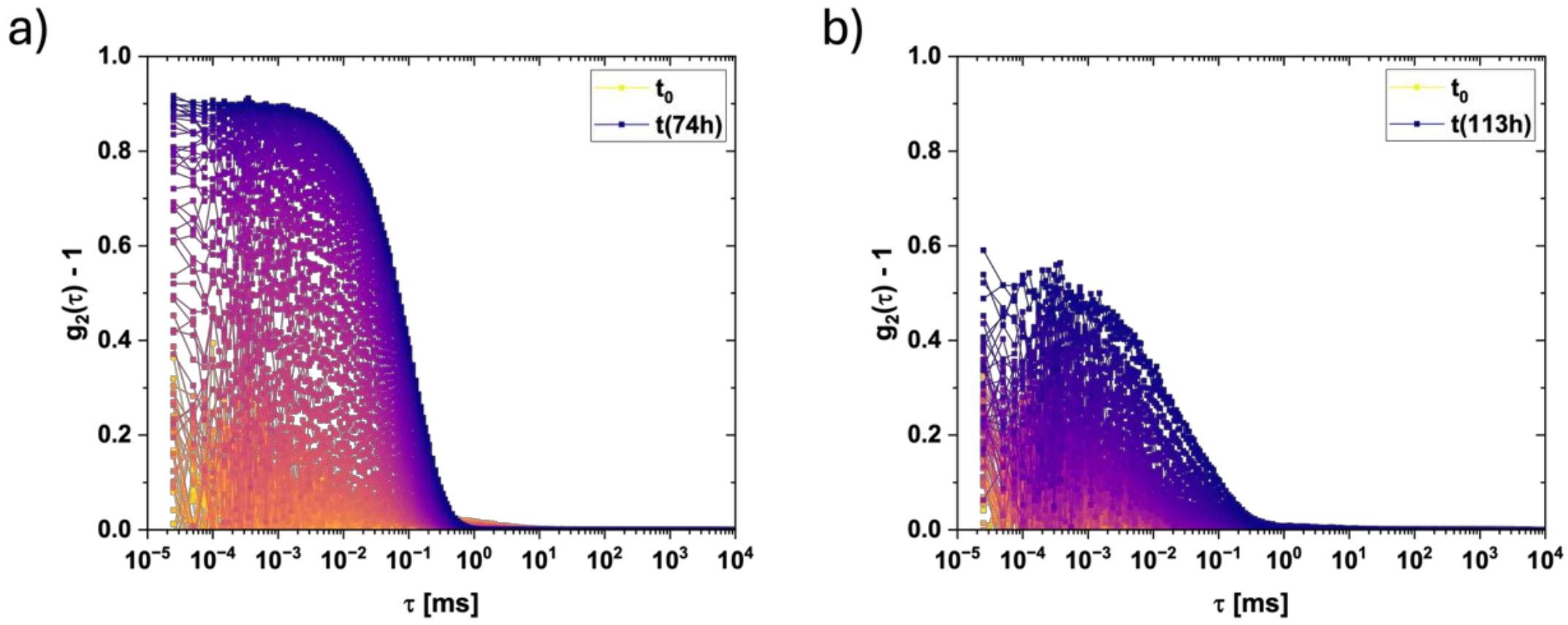

Figure S3. Correlation function of multiple DLS measurements during the formation of particles in the ternary mixture H_2O / EtOH / TEOS at point C (a) without the addition of a catalyst and (b) with arginine as catalyst (b). The gradient from yellow (t_0) to purple (t_{end}) visualizes the change of the samples over time. The hydrolysis ratio R = 92.5 and the solvent ratio S = 1.7.

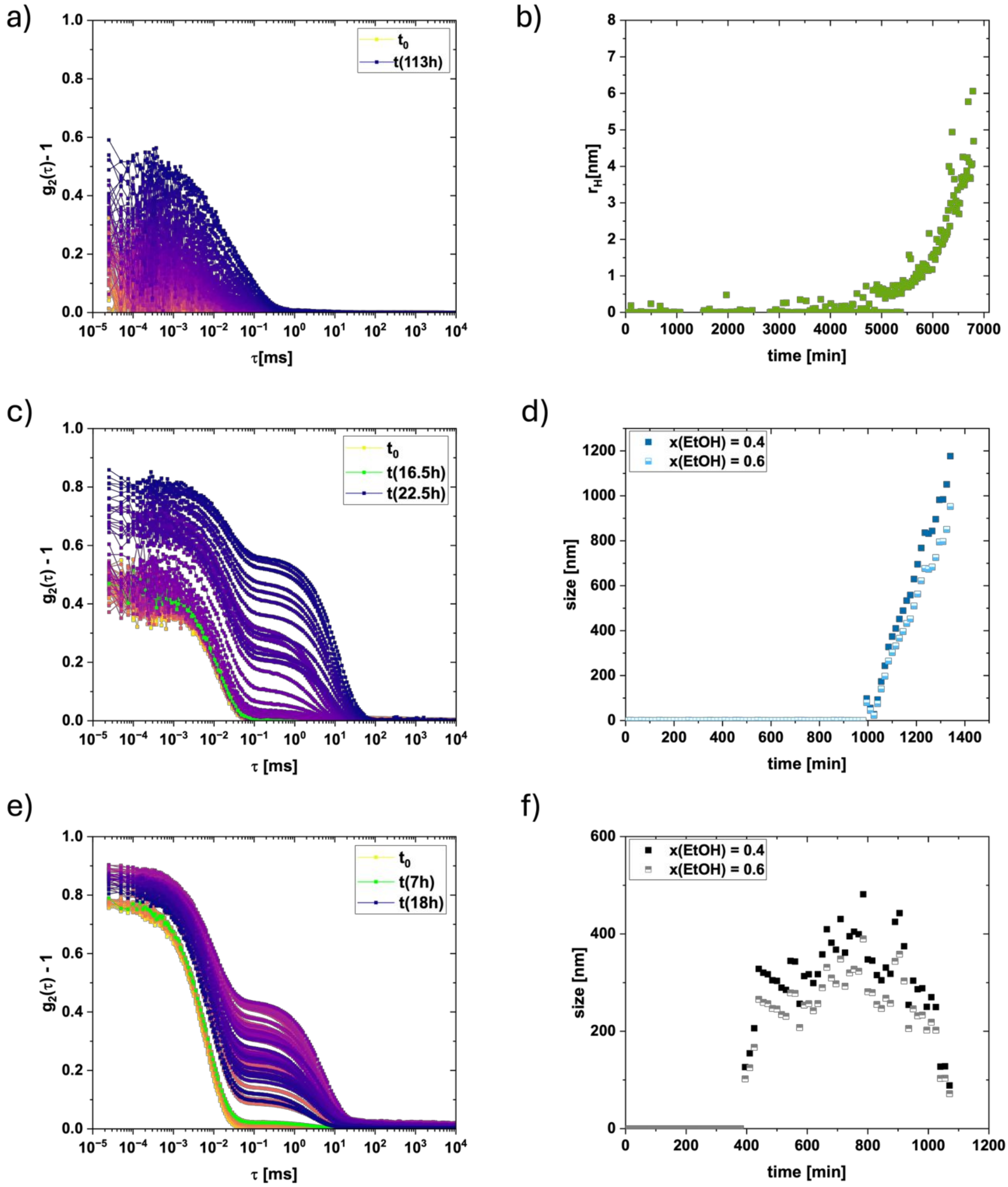

Figure S4. Correlation function of multiple DLS measurements during the formation of particles in the ternary mixtures (a) H_2O / EtOH / TEOS at point C, (c) H_2O / EtOH / 1-octanol + TEOS at point g and (e) H_2O / EtOH / toluene + TEOS at point f with the addition of arginine as catalyst. The gradient from yellow (t_0) to purple (t_{end}) visualizes the change of the samples over time. The respective change in hydrodynamic radius over time is shown in (b), (d), and (f).

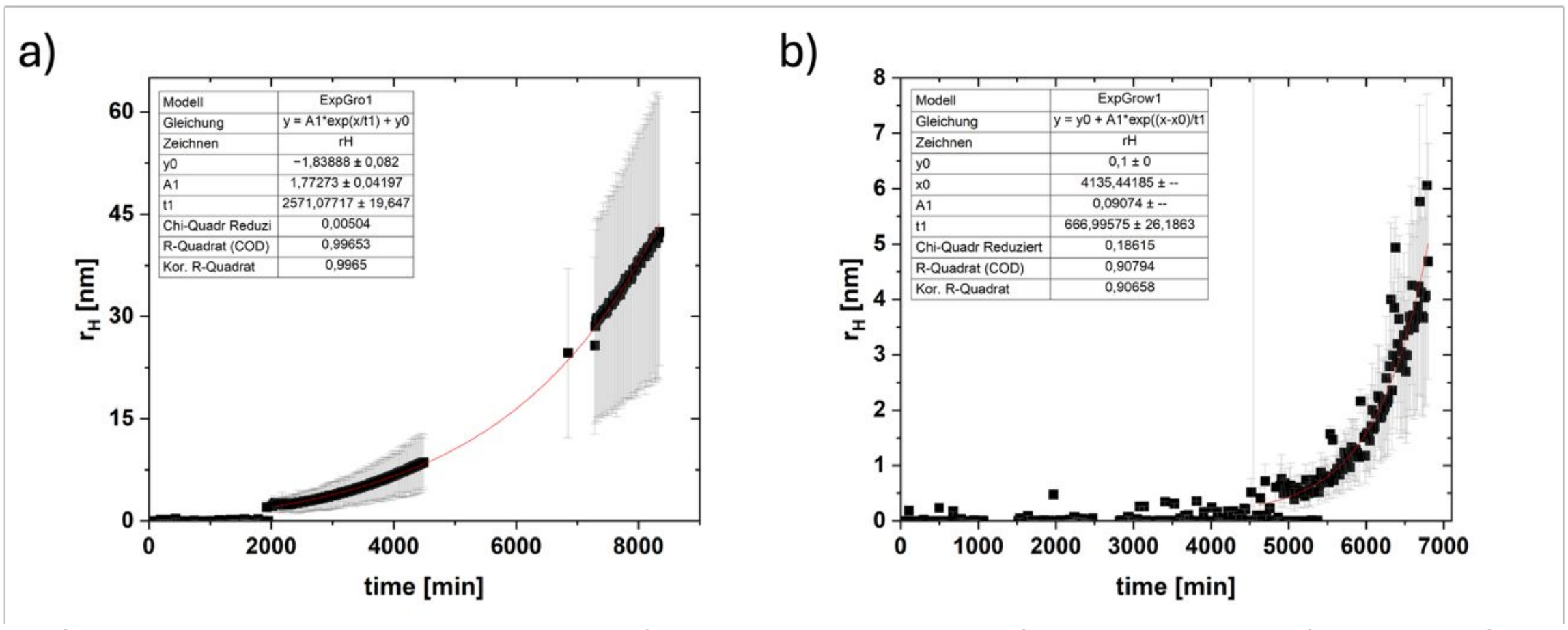

Figure S5. Hydrodynamic radius (r_H) derived from time dependent DLS measurements of the point C in the H_2O / EtOH / TEOS ternary mixture with (a) no catalyst and (b) arginine as catalyst. The corresponding correlation functions can be found in Figure S3a and Figure S3b, respectively.

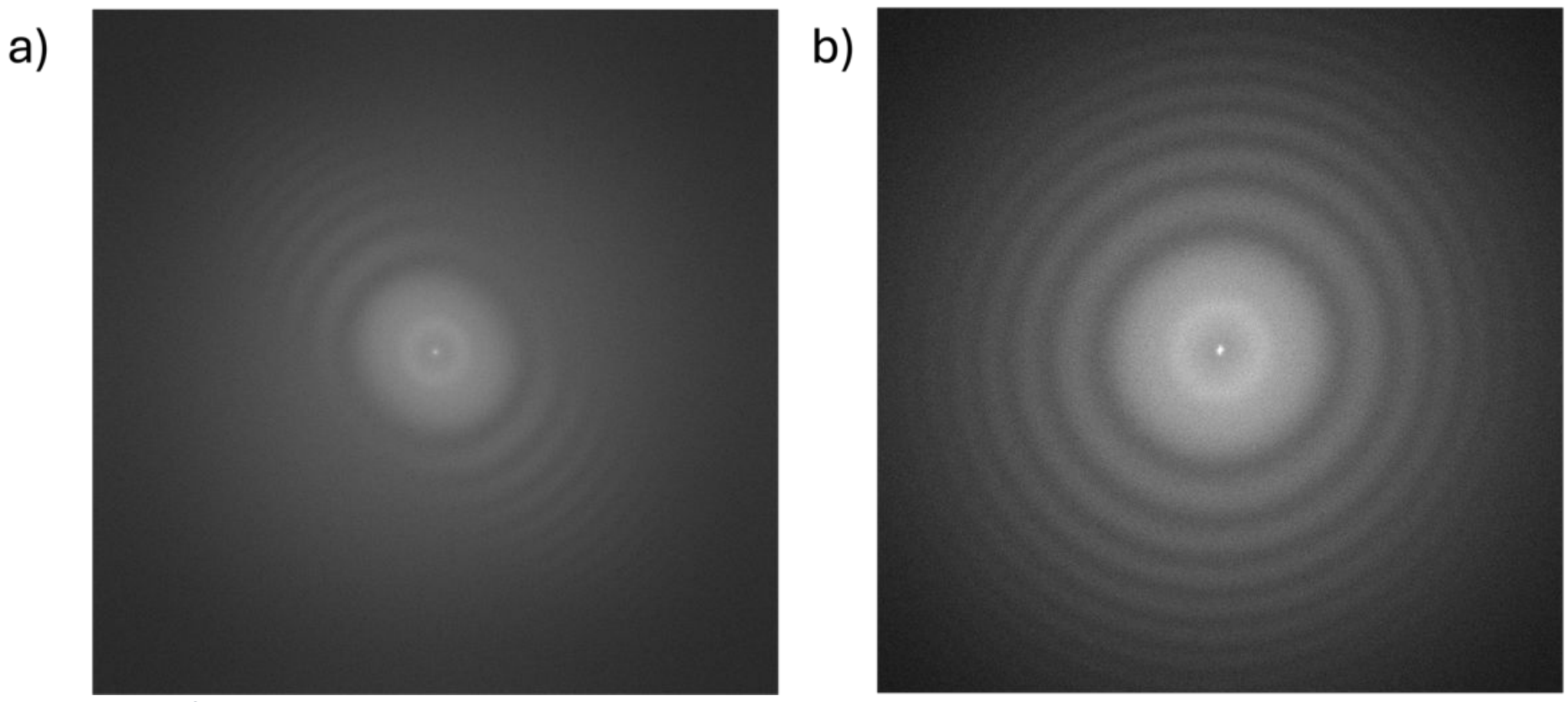

Figure S6. (a) FFT of the micrograph shown in Figure 8a. The white circle corresponds to the distance between individual particles giving an estimate for their size between 5.51 nm and 12.81 nm after radial integration. (b) FFT of the micrograph shown in Figure 8c. The white circle corresponds to the distance between individual particles giving an estimate for their size between 3.53 nm and 7.43 nm after radial integration.

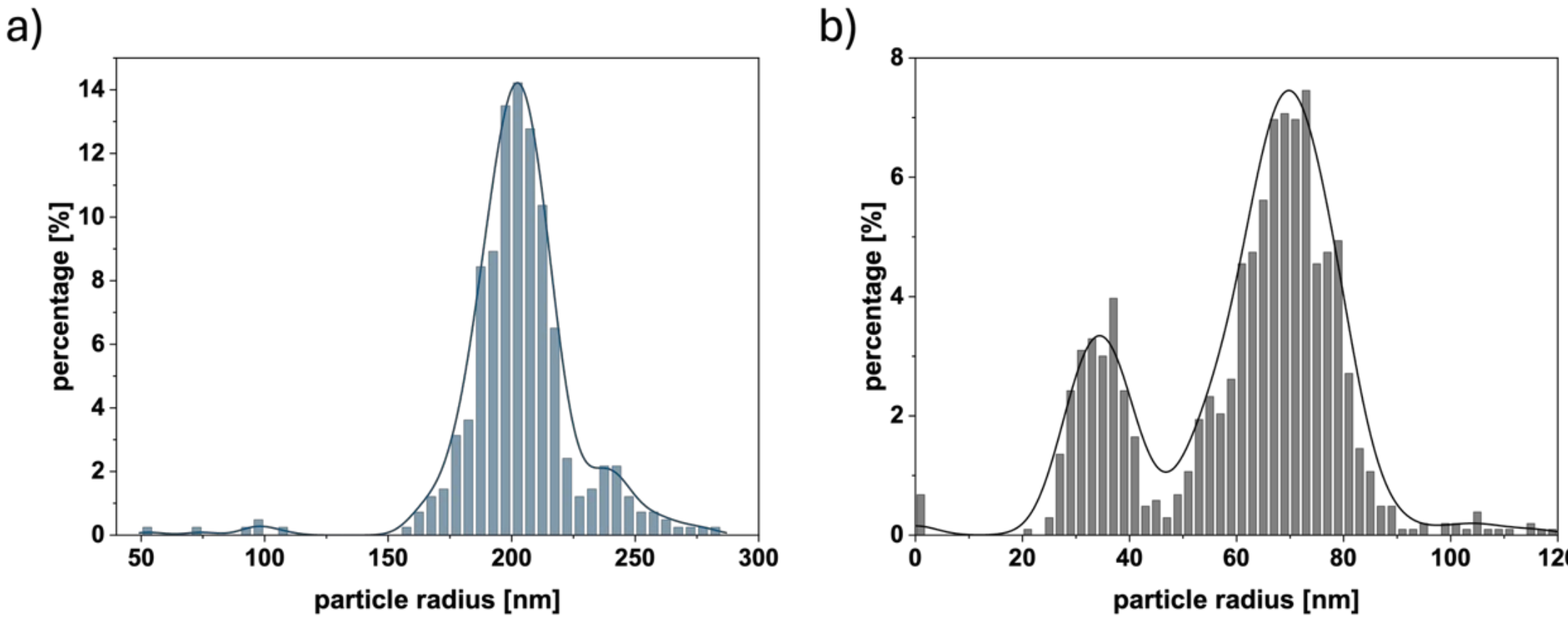

Figure S7. Histograms of measured particles from TEM micrographs. (a) 415 particles were measured for g and (b) 1026 particles were measured for f. Corresponding TEM micrographs can be found in Figure 8b and Figure 8d.

Table S5. Hydrodynamic radii of particles synthesized in the different systems. Values derived from DLS measurements and calculated from distribution fits of the histograms or are derived from the FFTs of the respective

micrographs (cf. Figure S8 and Figure S9). x(EtOH) was estimated to be around 0.6 for all samples except C. For C the composition of a fully hydrolyzed solution was used with x(EtOH) = 0.37.

	Derived from DLS			calculated		
	r_H [nm]	width [nm]	PD.I.	r_H [nm]	width [nm]	PD.I.
C	2.91	1.28	0.194	4.58	2.15	0.111
f	55.95	33.17	0.352	57.49	43.09	0.101
g	273.41	129.48	0.224	207.12	42.49	0.008
j	2.43	0.61	0.062	2.74	1.15	0.032

Table S6. Estimated TEOS-to-silica conversion, resulting silica mass, and particle number for the systems C, f, g, and j, calculated from the weighed-in TEOS amounts given in Section 2.6 and the particle radii r given in Table S5. The conversion was assumed to range from 92.7% to the theoretical maximum of 100%, and the silica particle density was assumed to be 2.07 g/cm³. [2]

point	system	m(TEOS) [mg]	m(SiO_2) [mg]	N [NP/mL]
C	TEOS	238	63.7 - 68.7	$1.43 - 1.54*10^{16}$
f	Toluene	115	30.7 - 33.1	$5.20 - 5.61*10^{12}$
g	1-Octanol	164	43.9 - 47.4	$1.58 - 1.71*10^{11}$
j	1-Octanol	95	25.3 - 27.3	$2.67 - 2.88*10^{16}$

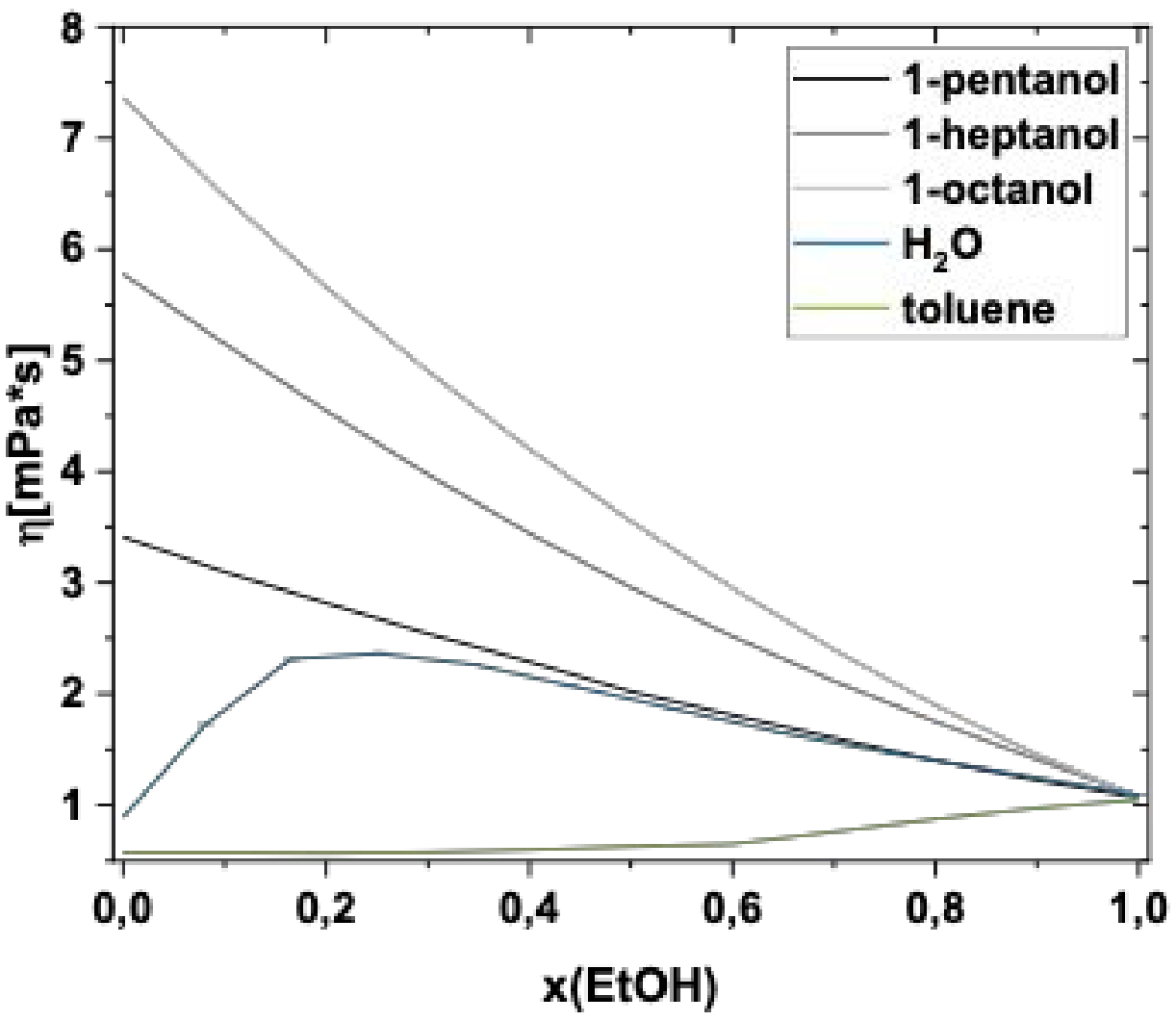

Figure S8. Viscosities of various binary mixtures of ethanol and the used solvents in the SFME systems as functions of the molar fraction of ethanol (x(EtOH)). While the value of the water ethanol mixture was measured, the others were taken from literature. [3–6]

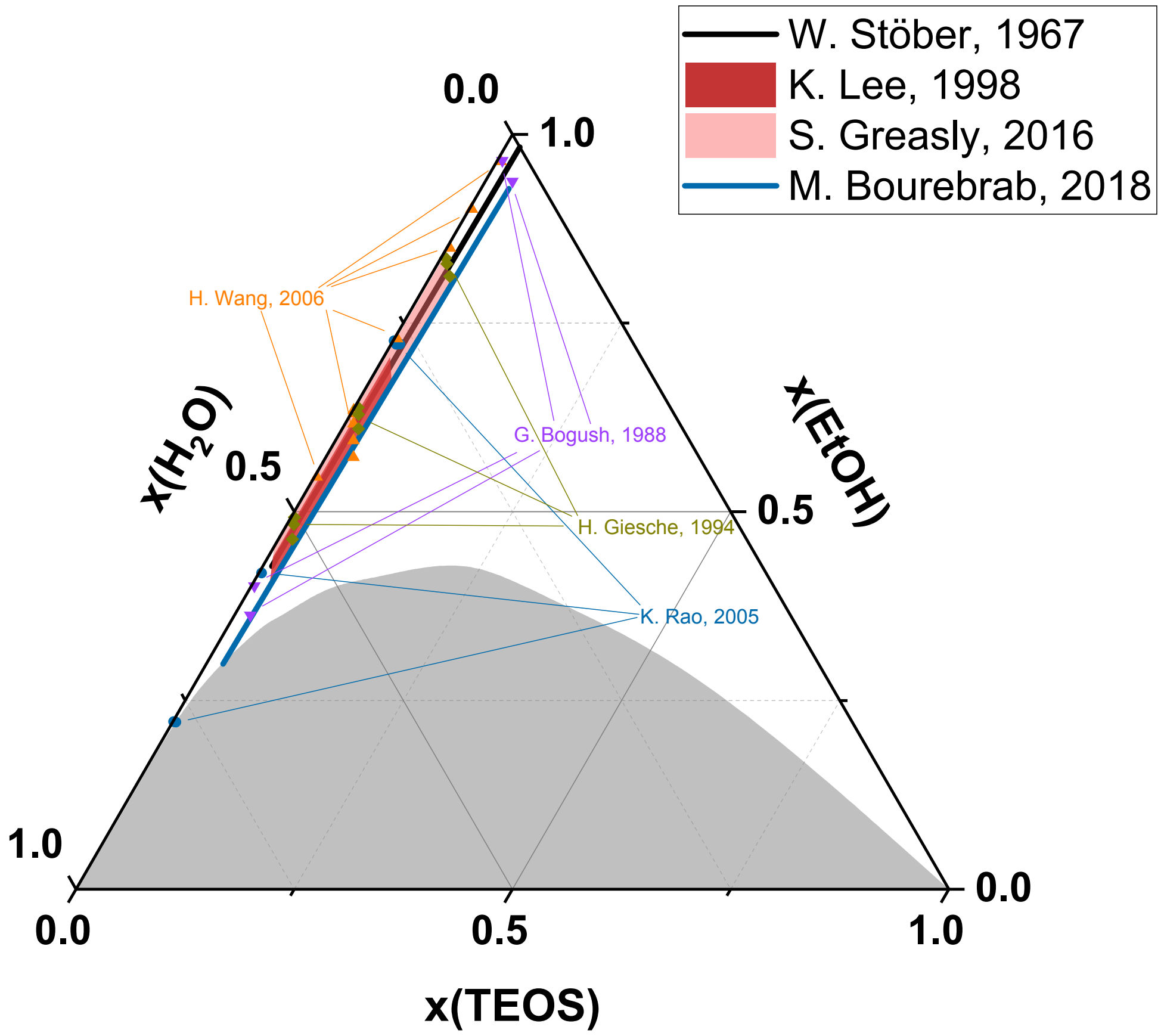

Figure S9. Phase diagrams of the ternary mixture of H_2O / EtOH / TEOS expressed in molar fractions x. The positions in the ternary space of relevant publications on Stöber syntheses were plotted. The highlighted areas correspond to screenings conducted by [7–10]. The single points were chosen as all references investigated rather different concentration ranges. [2,11–13]

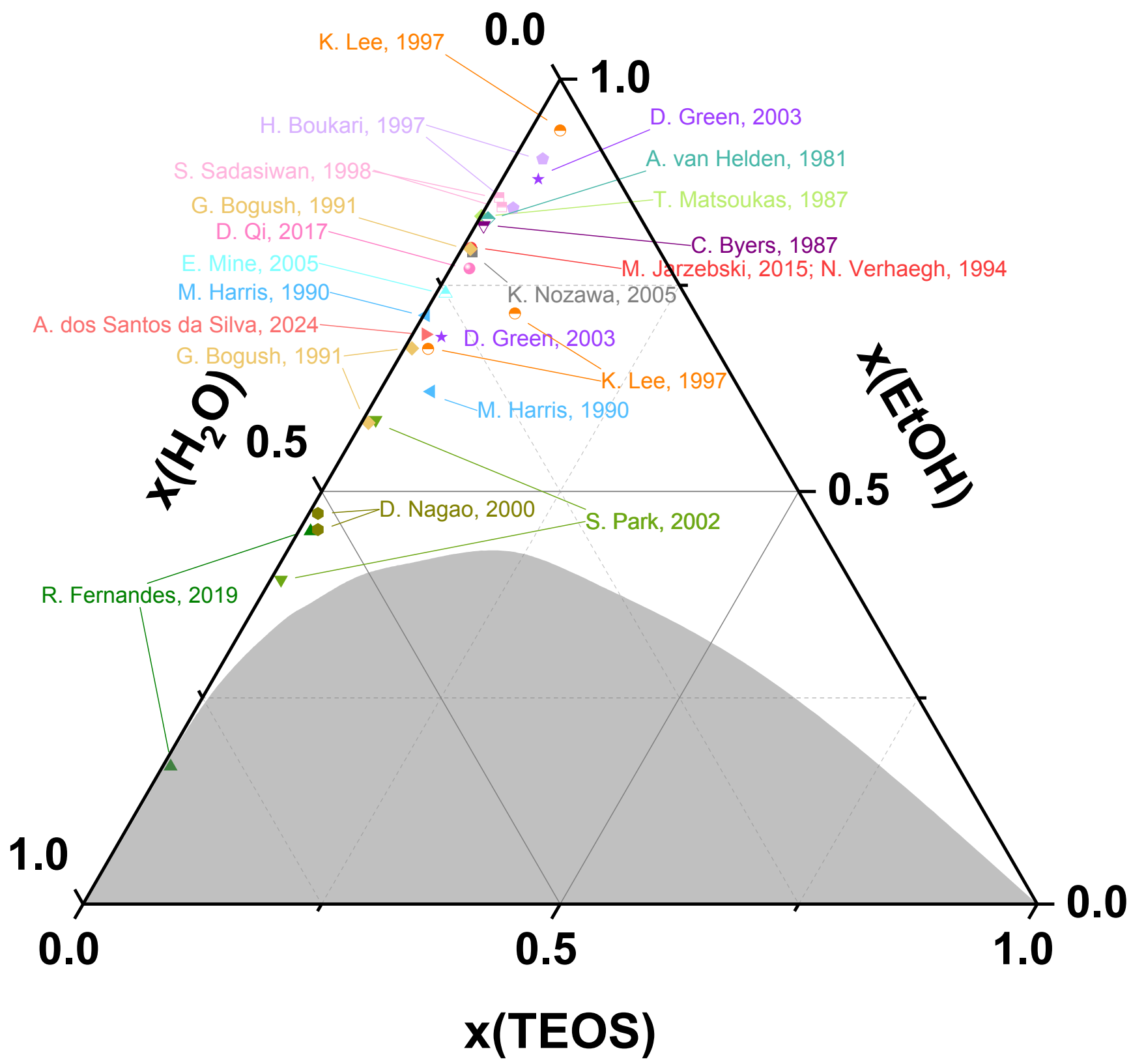

Figure S10. Phase diagram of the ternary mixture of H_2O / EtOH / TEOS expressed in molar fractions x. The positions in the ternary space of relevant publications on Stöber syntheses were plotted. The shown references only conducted experiments on few points within the ternary phase diagram [8,14–31] Note that a majority of the conducted experiments are at reduced water concentrations.

References

(1) Rohr, L.; Denk, P.; Blahnik, J.; Müller, E.; Sztucki, M.; Kunz, W. The Influence of Surface-Active Compounds on Surfactant-Free Microemulsions. *Aggregate, submitted* **2026**.

(2) Bogush, G. H.; Tracy, M. A.; Zukoski, C. F. Preparation of Monodisperse Silica Particles: Control of Size and Mass Fraction. *J. Non. Cryst. Solids* **1988**, *104* (1), 95–106. https://doi.org/10.1016/0022-3093(88)90187-1

(3) Cano-Gómez, J. J.; Iglesias-Silva, G. A.; Ramos-Estrada, M.; Hall, K. R. Densities and Viscosities for Binary Liquid Mixtures of Ethanol + 1-Propanol, 1-Butanol, and 1-Pentanol from (293.15 to 328.15) K at 0.1 MPa. *J. Chem. Eng. Data* **2012**, *57* (9), 2560–2567. https://doi.org/10.1021/je300632p

(4) Nikam, P. S.; Jagdale, B. S.; Sawant, A. B.; Hasan, M. Densities and Viscosities of Binary Mixtures of Toluene with Methanol, Ethanol, Propan-1-Ol, Butan-1-Ol, Pentan-1-Ol, and 2-Methylpropan-2-Ol at (303.15, 308.15, 313.15) K. *J. Chem. Eng. Data* **2000**, *45* (4), 559–563. https://doi.org/10.1021/je990317i

(5) Rauf, M. A.; Stewart, G. H.; Farhataziz. Viscosities and Densities of Binary Mixtures of 1-Alkanois from 15 to 55 °C. *J. Chem. Eng. Data* **1983**, *28* (3), 324–328. https://doi.org/10.1021/je00033a012

(6) Shan, Z.; Asfour, A. F. A. Viscosities and Densities of Nine Binary 1-Alkanol Systems at 293.15 K and 298.15 K. *J. Chem. Eng. Data* **1999**, *44* (1), 118–123. https://doi.org/10.1021/je980177x

(7) Stöber, W.; Fink, A.; Bohn, E. Controlled Growth of Monodisperse Silica Spheres in the Micron Size Range. *J. Colloid Interface Sci.* **1968**, *26* (1), 62–69. https://doi.org/10.1016/0021-9797(68)90272-5

(8) Lee, K.; Sathyagal, A. N.; McCormick, A. V. A Closer Look at an Aggregation Model of the Stöber Process. *Colloids Surf. A Physicochem. Eng. Asp.* **1998**, *144* (1–3), 115–125. https://doi.org/10.1016/S0927-7757(98)00566-4

(9) Greasley, S. L.; Page, S. J.; Sirovica, S.; Chen, S.; Martin, R. A.; Riveiro, A.; Hanna, J. V.; Porter, A. E.; Jones, J. R. Controlling Particle Size in the Stöber Process and Incorporation of Calcium. *J. Colloid Interface Sci.* **2016**, *469*, 213–223. https://doi.org/10.1016/j.jcis.2016.01.065

(10) Bourebrab, M. A.; Oben, D. T.; Durand, G. G.; Taylor, P. G.; Bruce, J. I.; Bassindale, A. R.; Taylor, A. Influence of the Initial Chemical Conditions on the Rational Design of Silica Particles. *J. Solgel Sci. Technol.* **2018**, *88* (2), 430–441. https://doi.org/10.1007/s10971-018-4821-9

(11) Wang, H.-C.; Wu, C.-Y.; Chung, C.-C.; Lai, M.-H.; Chung, T.-W. Analysis of Parameters and Interaction between Parameters in Preparation of Uniform Silicon Dioxide Nanoparticles Using Response Surface Methodology. *Ind. Eng. Chem. Res.* **2006**, *45* (24), 8043–8048. https://doi.org/10.1021/ie060299f

(12) Giesche, H. Synthesis of Monodispersed Silica Powders I. Particle Properties and Reaction Kinetics. *J. Eur. Ceram. Soc.* **1994**, *14* (3), 189–204. https://doi.org/10.1016/0955-2219(94)90087-6

(13) Rao, K. S.; El-Hami, K.; Kodaki, T.; Matsushige, K.; Makino, K. A Novel Method for Synthesis of Silica Nanoparticles. *J. Colloid Interface Sci.* **2005**, *289* (1), 125–131. https://doi.org/10.1016/j.jcis.2005.02.019
(14) Boukari, H.; Lin, J. S.; Harris, M. T. Small-Angle X-Ray Scattering Study of the Formation of Colloidal Silica Particles from Alkoxides: Primary Particles or Not? *J. Colloid Interface Sci.* **1997**, *194* (2), 311–318. https://doi.org/10.1006/jcis.1997.5112
(15) Sadasivan, S.; Dubey, A. K.; Li, Y.; Rasmussen, D. H. Alcoholic Solvent Effect on Silica Synthesis—NMR and DLS Investigation. *J. Solgel Sci. Technol.* **1998**, *12* (1), 5–14. https://doi.org/10.1023/A:1008659708390
(16) Bogush, G. H.; Zukoski, C. F. Studies of the Kinetics of the Precipitation of Uniform Silica Particles through the Hydrolysis and Condensation of Silicon Alkoxides. *J. Colloid Interface Sci.* **1991**, *142* (1), 1–18. https://doi.org/10.1016/0021-9797(91)90029-8
(17) Bogush, G. H.; Zukoski, C. F. Uniform Silica Particle Precipitation: An Aggregative Growth Model. *J. Colloid Interface Sci.* **1991**, *142* (1), 19–34. https://doi.org/10.1016/0021-9797(91)90030-C
(18) Qi, D.; Lin, C.; Zhao, H.; Liu, H.; Lü, T. Size Regulation and Prediction of the SiO 2 Nanoparticles Prepared via Stöber Process. *J. Dispers. Sci. Technol.* **2017**, *38* (1), 70–74. https://doi.org/10.1080/01932691.2016.1143373
(19) Mine, E.; Nagao, D.; Kobayashi, Y.; Konno, M. Solvent Effects on Particle Formation in Hydrolysis of Tetraethyl Orthosilicate. *J. Solgel Sci. Technol.* **2005**, *35* (3), 197–201. https://doi.org/10.1007/s10971-005-2289-x
(20) Harris, M. T.; Brunson, R. R.; Byers, C. H. The Base-Catalyzed Hydrolysis and Condensation Reactions of Dilute and Concentrated TEOS Solutions. *J. Non. Cryst. Solids* **1990**, *121* (1–3), 397–403. https://doi.org/10.1016/0022-3093(90)90165-I
(21) dos Santos da Silva, A.; dos Santos, J. H. Z. Stöber Method and Its Nuances over the Years. *Adv. Colloid Interface Sci.* **2023**, *314* (March), 102888. https://doi.org/10.1016/j.cis.2023.102888
(22) Fernandes, R. S.; Raimundo, I. M.; Pimentel, M. F. Revising the Synthesis of Stöber Silica Nanoparticles: A Multivariate Assessment Study on the Effects of Reaction Parameters on the Particle Size. *Colloids Surf. A Physicochem. Eng. Asp.* **2019**, *577* (January), 1–7. https://doi.org/10.1016/j.colsurfa.2019.05.053
(23) Green, D. L.; Lin, J. S.; Lam, Y.-F.; Hu, M. Z.-C.; Schaefer, D. W.; Harris, M. T. Size, Volume Fraction, and Nucleation of Stober Silica Nanoparticles. *J. Colloid Interface Sci.* **2003**, *266* (2), 346–358. https://doi.org/10.1016/S0021-9797(03)00610-6
(24) Van Helden, A. K.; Jansen, J. W.; Vrij, A. Preparation and Characterization of Spherical Monodisperse Silica Dispersions in Nonaqueous Solvents. *J. Colloid Interface Sci.* **1981**, *81* (2), 354–368. https://doi.org/10.1016/0021-9797(81)90417-3
(25) Matsoukas, T.; Gulari, E. Dynamics of Growth of Silica Particles from Ammonia-Catalyzed Hydrolysis of Tetra-Ethyl-Orthosilicate. *J. Colloid Interface Sci.* **1988**, *124* (1), 252–261. https://doi.org/10.1016/0021-9797(88)90346-3

(26) Byers, C. H.; Harris, M. T.; Williams, D. F. Controlled Microcrystalline Growth Studies by Dynamic Laser-Light-Scattering Methods. *Ind. Eng. Chem. Res.* **1987**, *26* (9), 1916–1923. https://doi.org/10.1021/ie00069a033

(27) Jarzębski, M.; Śliwa, T.; Jarzębska, M.; Szutkowski, K. Fabrication of Size-Tunable Silica Particles During Seed-Growth Process. *Current Topics in Biophysics* **2015**, *37* (1), 35–41. https://doi.org/10.2478/ctb-2014-0072

(28) Verhaegh, N. A. M.; Van Blaaderen, A. Dispersions of Rhodamine-Labeled Silica Spheres: Synthesis, Characterization, and Fluorescence Confocal Scanning Laser Microscopy. *Langmuir* **1994**, *10* (5), 1427–1438. https://doi.org/10.1021/la00017a019

(29) Nozawa, K.; Gailhanou, H.; Raison, L.; Panizza, P.; Ushiki, H.; Sellier, E.; Delville, J. P.; Delville, M. H. Smart Control of Monodisperse Stöber Silica Particles: Effect of Reactant Addition Rate on Growth Process. *Langmuir* **2005**, *21* (4), 1516–1523. https://doi.org/10.1021/la048569r

(30) Nagao, D.; Satoh, T.; Konno, M. A Generalized Model for Describing Particle Formation in the Synthesis of Monodisperse Oxide Particles Based on the Hydrolysis and Condensation of Tetraethyl Orthosilicate. *J. Colloid Interface Sci.* **2000**, *232* (1), 102–110. https://doi.org/10.1006/jcis.2000.7195

(31) Park, S. K.; Kim, K. Do; Kim, H. T. Preparation of Silica Nanoparticles: Determination of the Optimal Synthesis Conditions for Small and Uniform Particles. *Colloids Surf. A Physicochem. Eng. Asp.* **2002**, *197* (1–3), 7–17. https://doi.org/10.1016/S0927-7757(01)00683-5
